# Targeted next-generation sequencing as a comprehensive test for Mendelian diseases: a cohort diagnostic study

**DOI:** 10.1038/s41598-018-30151-z

**Published:** 2018-08-03

**Authors:** Yan Sun, Jianfen Man, Yang Wan, Gao Pan, Lique Du, Long Li, Yun Yang, Liru Qiu, Qing Gao, Handong Dan, Liangwei Mao, Zhengyu Cheng, Chen Fan, Jing Yu, Mufei Lin, Karsten Kristiansen, Yin Shen, Xiaoming Wei

**Affiliations:** 1 Department of Biology, University of Copenhagen, Copenhagen, DK-2200 Denmark; 2 BGI Genomics, BGI-Shenzhen, Shenzhen, 518083 China; 3 BGI-Wuhan, BGI-Shenzhen, Wuhan, 430074 China; 4 Fuyang People’s Hospital, Fuyang, 236000 China; 5 The Nephrology Division of Department of Pediatrics, Tongji Hospital, Tongji Medical College, Huazhong University of Science and Technology, Wuhan, 430030 China; 6 Eye Center, Renmin Hospital of Wuhan University, Wuhan University, Wuhan, 430060 China; 7 China National GeneBank, BGI-Shenzhen, Shenzhen, 518120 China

## Abstract

With the development of next generation sequencing, more and more common inherited diseases have been reported. However, accurate and convenient molecular diagnosis cannot be achieved easily because of the enormous size of disease causing mutations. In this study, we introduced a new single-step method for the genetic analysis of patients and carriers in real clinical settings. All kinds of disease causing mutations can be detected at the same time in patients with Mendelian diseases or carriers. First, we evaluated this technology using YH cell line DNA and 9 samples with known mutations. Accuracy and stability of 99.80% and 99.58% were achieved respectively. Then, a total of 303 patients were tested using our targeted NGS approaches, 50.17% of which were found to have deleterious mutations and molecular confirmation of the clinical diagnosis. We identified 219 disease causing mutations, 43.84% (96/219) of which has never been reported before. Additionally, we developed a new deleteriousness prediction method for nonsynonymous SNVs, and an automating annotation and diagnosis system for Mendelian diseases, thus greatly assisting and enhancing Mendelian diseases diagnosis and helping to make a precise diagnosis for patients with Mendelian diseases.

## Introduction

Mendelian diseases are a series of diseases following the principles of Mendelian inheritance. So far, more than 8,000 Mendelian inheritance diseases have been included in Online Mendelian Inheritance in Man (the OMIM database, www.omim.org). Many methods have been performed for the diagnosis of Mendelian diseases in clinical practice, for example, Sanger sequencing of known disease causing genes. As is known, Sanger sequencing is widely considered as the gold standard for sequencing, however, there are some drawbacks in this technology: laborious, expensive, and time consuming.

With the rapid development of next generation sequencing (NGS) technology, all kinds of diseasing causing mutations, such as SNVs (single nucleotide variants), Indel (small insertion/deletion) and CNVs (copy number variants), can be detected at the same time in patients with Mendelian diseases or carriers^1^. It has been proved that NGS based technology is a powerful tool for the detection of pathogenic mutations, especially in patients with monogenic disorders^2^. Researchers performed NGS to analyze tens to thousands of genes simultaneously in a single assay^1,3,4^. This makes NGS more suitable in the diagnosis of Mendelian diseases, and highly improved the diagnostic yield^5^. Comprehensive characterization of genetic diseases using NGS technology has become an option for researchers and clinicians. However, annotation and interpretation of NGS data is tedious and time-consuming, and require highly specific expertise in this filed. Our new method introduced here can help clinical scientists to efficiently integrate all their data more quickly and form a whole picture of the detected variants.

WGS (whole genome sequencing) and WES (Whole exome sequencing) have been applied in the diagnostic of Mendelian genetic disorders or the screening of carriers^6–9^. However, it is still a major challenge to detect few disease causing mutations in the vast potential variants in human genome^10^. Targeted NGS is recognized as a cost effective method for the diagnostic of Mendelian genetic disorders^1,3,4^. Targeted NGS with high throughput and lower cost can target disease related regions in human genome more efficiently, and detect variants more sensitively. This technology has been widely used in the screening of mutations and effective diagnosis of genetic diseases in clinical setting^11–14^. What is more, targeted NGS targets a set of genes related to specific disease phenotypes, which could result in a higher sequencing coverage. Variant calling is more accurate for region of interest when performing targeted NGS^15^.

In this study, we designed a chip (array based) containing 4,689 nuclear genes related to Mendelian diseases, and tested its performance in detecting clinical relevant mutations using HiSeq platform. Then, we applied this panel to 303 clinical cases, and identified disease causing mutations in 152 patients. What is more, we developed a new deleteriousness prediction method for nonsynonymous SNVs, and an automating annotation and diagnosis system for Mendelian diseases. It can greatly assist and enhance Mendelian diseases diagnosis and help to make a precise diagnosis for the patients.

## Results

### Evaluation of accuracy and stability

Before applying this technology to real clinical patients, we evaluated the accuracy and stability of targeted NGS as reported before^1^. To assess the accuracy of this technology, we compared the SNPs of a YH cell line sample (C1) detected using targeted NGS with the genotyping results detected using Illumina’s Human Zhonghua-8 Bead Chips (SNP Array). The analysis of C1 using SNP Array was carried out blindly. In C1, there are a total of 4416 SNPs detected in the region of the chip (10,258,198 bp). In the selected loci, 99.80% (4,407/4,416) genotypes detected by targeted NGS in sample C1 were accordant to the results detected by SNP Array, which showed a high accuracy of our method. We also screened the 4,689 nuclear genes in 9 probands with known mutations using targeted NGS to evaluate the technology (Table 1). Sanger sequencing and real-time PCR validation of the known mutations in the 9 probands had been completed prior to this study. A total of 11 known mutations were detected in the 9 probands using targeted NGS technology. The results are exactly the same as obtained using real-time PCR and Sanger sequencing, indicating complete consistency of the methods. These results indicated that targeted NGS technology used in this study provides high accuracy.

**Table 1:** Nine probands with known mutations.

Proband	Disease	Gene	Mutation	Validation
P1	Dent Disease 1	CLCN5	c.778_798delACTCTG GTTATCAAAACCATC	Sanger
P2	X-Linked Ichthyosis	STS	Whole gene deletion	QPCR
P3	Mucolipidosis II/III Alpha&Beta	GNPTAB	c.1090 C > T; c.2404 C > T	Sanger
P4	Charcot-Marie-Tooth disease	PMP22	c.215 C > T	Sanger
P5	Polycystic kidney disease 1	PKD1	c.8835 C > G	Sanger
P6	Duchenne/Becker Muscular Dystrophy	DMD	CDS 10–12 deletion	QPCR
P7	Nephrotic Syndrome Type 2	NPHS2	c.593 A > C; c.538 G > A	Sanger
P8	Pseudohypoaldosteronism Type IB	SCNN1B	c.1853C > G	Sanger
P9	Charcot-Marie-Tooth disease	PMP22	Whole gene duplication	QPCR

To assess the stability of the technology, YH cell line were sequenced three times in different batches (C1, C2 and C3). There are 10,133,027 genotype can be detected in the 3 YH cell line samples. We then compared all the genotypes detected in C1, C2 and C3. The results showed that the proportion of the same genotype detected is 99.58% (10,090,060/10,133,027) in different batches, which indicated high stability of this technology.

### Targeted NGS approach

In this study, we developed a targeted NGS assay to detect disease causing alterations in 303 patients diagnosed with Mendelian diseases using HiSeq platform.

A total of 303 patients were recruited in this study. Using targeted NGS, we obtained high-quality reads of all the samples. After base calling and image analysis using Illumina HiSeq sequencers, primary data was received in the form of FASTQ. Then we performed a new bioinformatics analysis pipeline to analyze the raw sequencing data. The sequencing results of all the patients are as follows: more than 99.55% coverage of the target region was achieved for each sample, and an average sequencing depth of 250.82-fold was achieved for each sample.

### Deleteriousness prediction for nonsynonymous SNVs

In order to attain the best classifier for rare missense variants in human genome, we developed a new pathogenicity prediction method (MPPS, Mutation Pathogenicity Prediction Software) in this study. MPPS combined a total of 17 current deleteriousness-scoring methods, including 8 functional prediction scores (Polyphen2_HDIV^16^, Polyphen2_HVAR^16^, MutationTaster^17^, SIFT^18^, LRT^19^, FATHMM^20^, MutationAssessor^21^ and M-CAP^22^), 7 new prediction scores (PROVEAN^23^, VEST3^24^, MetaSVM^25^, MetaLR^25^, fathmm-MKL^26^, CADD^27^ and DANN^28^) and 2 conservation scores (GERP++^29^ and phyloP100way^30^).

**Figure 1:**
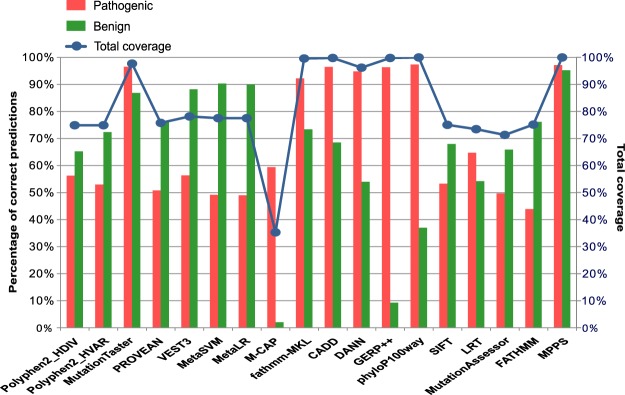
Accuracy and coverage of 17 prediction programs.

In the present study, we evaluated the performance of the 17 deleteriousness prediction methods and MPPS (Fig. 1). We collected a total of 25,220 known pathogenic variants from ClinVar (www.ncbi.nlm.nih.gov/clinvar), and 22,885 neutral variants from ClinVar (4,308) and our local database (18,577). The best performing methods in this test were MPPS and MutationTaster. The percentage of correct predictions of MutationTaster is 96.56% for pathogenic variants and 86.84% for neutral variants respectively. MPPS was able to correctly predict 97.12% of credibly pathogenic mutations and 95.21% of the credibly benign variants. There was a noticeable problem with several of the deleteriousness prediction programs: the lack of output because of the method’ inability to predict pathogenicity for a specific variant^31^. So we introduced the concept of Prediction Coverage. Prediction Coverage is defined by dividing the number of predicted variants (correctly or in correctly) by the total number of variants evaluated. Prediction Coverage was used to evaluate if all the variants can be predicted with a precise result or not. As shown in Fig. 1, 100% of variants (48,105/48,105) were successfully covered by MPPS, which is even better than MutationTaster (97.73%). MPPS could also make a great contribution to the automating result interpretation and reporting system in the next step.

### Automating result interpretation and reporting system

**Figure 2:**
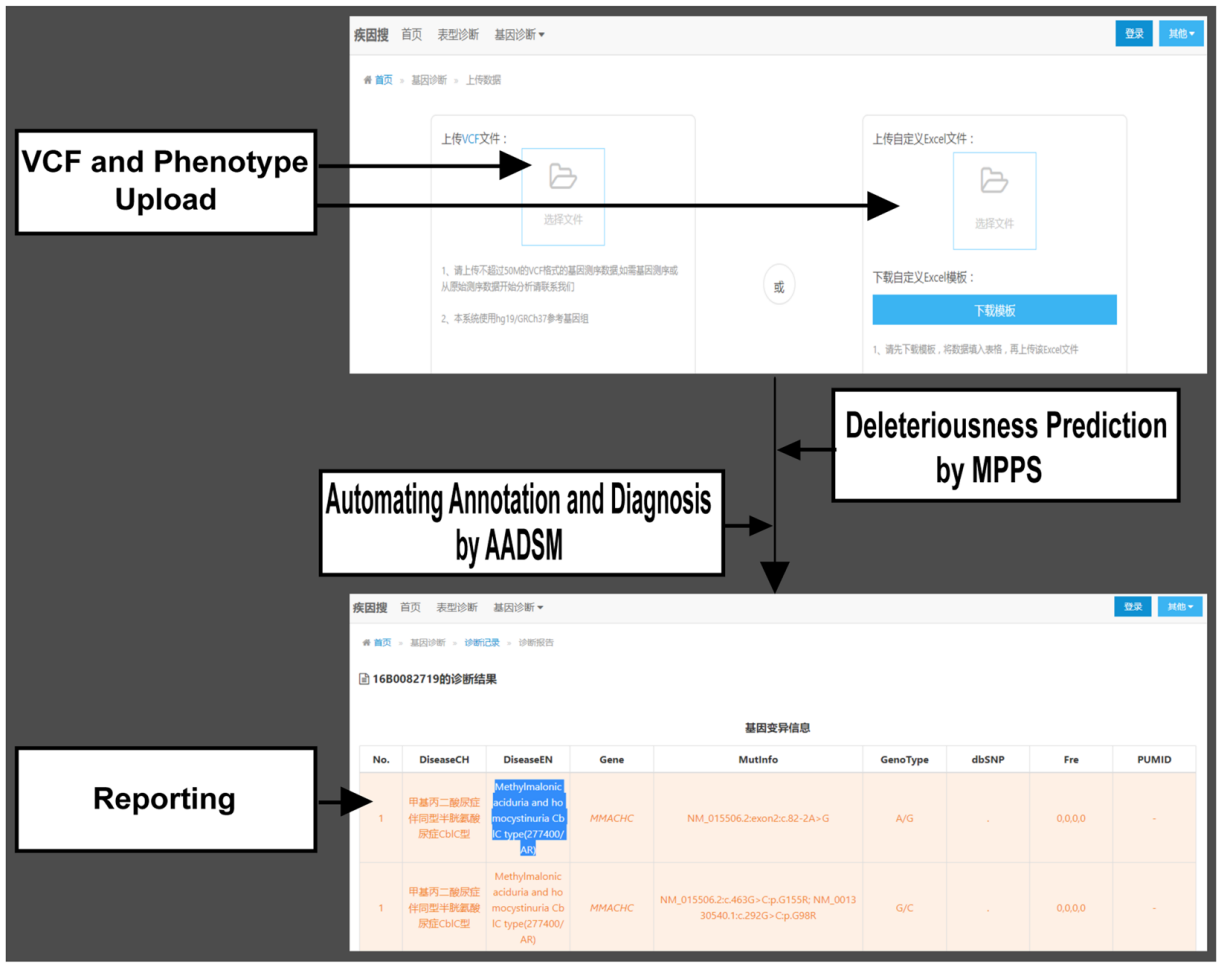
Automating annotation and diagnosis system for Mendelian disease (AADSM). AADSM is a web-based automating annotation and diagnosis system, which can assist and enhance diagnostics of Mendelian diseases.

In this study, we developed a web-based framework named AADSM (Automating Annotation and Diagnosis System for Mendelian disease) for automating analysis of clinical NGS data (Fig. 2). Clinical scientists can use this website to get information directly for diagnostics of Mendelian diseases by uploading the VCF (variant call format) file and the patients’ phenotypes.

Before analysis, we build our own gene-phenotype database and a scoring system. We use the gene-phenotype database and the scoring system to allow clinical researchers to carry out analysis of variant data, and help them to make a precise diagnosis for the patients. AADSM accepts variants input in the VCF formats. Patients’ phenotype also need to be upload before analysis (Fig. 2). The server performs a three step analysis process automatically, which mainly includes variant filtering and phenotype analysis, scoring, sorting and reporting. In the first step, all the variants will be filtered and annotated with our databases. At the same time, a gene list will be generated according to the clinical phenotype uploaded. In the second step, all the filtered mutations will be sorted using the scoring system we developed. The scoring system relies on the following rules: mutation frequency, mutation type, the consequence of a variant, and predicted categories from HGMD to determine the rank of a variant. In order to find the most related disease, the gene list generated in the first step will also be sorted using the scoring system. In the last step, a report will be automatically reported based on gene-disease relationship. The diseases and mutations with the highest score will be automated reported. Analysis results were presented in HTML pages, which contain all the details of annotation and the diagnosed diseases. These results can help clinical scientists to efficiently integrate variant related information and form a whole picture of their data more quickly.

To demonstrate the method’s diagnostic efficacy, we tested 56 patients in this study. A total of 85 disease causing mutations in the 56 patients were validated by methods other than NGS technology and identified previously by experienced researchers in this field. All the 56 patients were then diagnosed to be with specific Mendelian diseases according to the mutations identified. We also performed AADSM for automating analysis of clinical NGS data of the 56 patients. All the 85 disease causing mutations identified by experienced researchers were also detected by AADSM. In the 56 patients, the clearly diagnosed diseases in 32 patients (57.14%) ranked first in all the reported diseases, the clearly diagnosed diseases in 45 (80.36%) patients ranked top ten, and the clearly diagnosed diseases in 55 (98.21%) patients ranked top one hundred (Supplementary Material). These results can greatly assist and enhance Mendelian diseases diagnosis for clinical scientists and help to make a precise diagnosis for the patients.

### Identification and characterization of disease causing mutations

**Figure 3:**
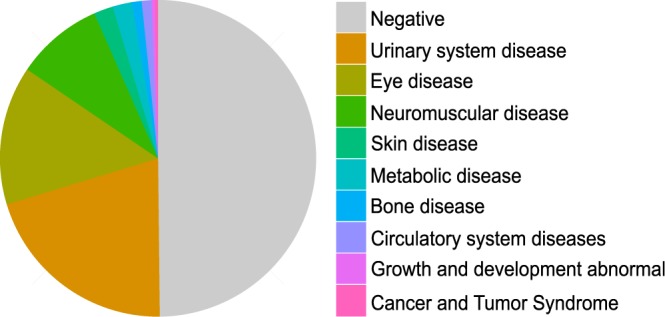
Positive patients found to have deleterious mutations and have molecular confirmation of the clinical diagnosis.

In this study, a total of 219 disease causing mutations were detected, including 129 missense, 26 nonsense, 33 Indel, 15 splicing and 16 CNV. 43.84% (96/219) of disease causing mutations has never been reported before (Supplementary Material). Of all the 303 random patients, 50.17% (152/303) were found to have deleterious mutations and have molecular confirmation of the clinical diagnosis (Fig. 3). The 152 patients belonged to 9 clinical classifications. It was also observed that Urinary system disease (20.46% of all the 152 positive patients), Eye disease (14.19%), and Neuromuscular disease (8.91%) were the most frequently detected clinical category in our cohort (Fig. 3). Cataract (22 patients), Alport Syndrome (16 patients), and Retinitis pigmentosa (10 patients) were found to contain the most patients (Supplementary Material). The results strongly suggested that targeted NGS technique is an informative and effective approach to make a precise diagnosis for patients with Mendelian diseases, and has great potential to be used in real clinical practice.

A total of 85 (38.81%) of identified pathogenic mutations were tested and confirmed by Sanger sequencing (Supplementary Material). Co-segregation analysis was also performed among some patients’ family members. The validation further confirmed the clinical diagnosis of the patients.

## Discussion

Accurate and timely detection of disease causing mutations in patients with Mendelian diseases plays a pivotal role in the era of personalized medicine. Because the involved genes number is large, traditional methods cannot be used to make a precise genetic diagnosis at a time^5^. With the rapid development of NGS, comprehensive characterization of genetic diseases using NGS technology has become an option for researchers and clinicians^3,4,6,11–14,32,33^. It has been proved that NGS based technology is a powerful tool for the detection of pathogenic mutations, especially in patients with Mendelian diseases.

In this study, we designed a new chip (array based) containing 4,689 genes related to Mendelian diseases, and performed targeted region enrichment and NGS for comprehensive mutation detection in 303 clinical cases. We reported disease causing mutations in 152 patients out of 303 random patients using targeted NGS. A total of 219 disease causing mutations (including 96 novel mutations) were identified. Some researchers reported that ~25% random patients were found to have deleterious mutations^8^. Based on our experience, the detection rate is highly dependent on the experience of the clinical scientist. In this study, all the potential patients were collected by experienced clinical scientists. Comparing to the results of previously publications, we included more genes related to Mendelian diseases, the diagnostic yield was also improved^1,3–5,8^.

To test the accuracy of this method, we compared the SNPs of a YH cell line sample detected using targeted NGS technology with the genotyping results detected using SNP Array. In the selected loci, 4,407 out of 4,416 genotypes detected by targeted NGS in sample C1 were accordant to the results detected by SNP Array, which means there are 9 genotypes inconsistent using targeted NGS and SNP Array. We did NOT validate these inconsistent genotypes in the present study because in our previous study^1^, we also found 5 inconsistent genotypes when accessing the accuracy of targeted NGS technology. It turns out that all the genotypes of inconsistent loci detected by targeted NGS were consistent with that of Sanger sequencing^1^, which suggested that our method could provide high accuracy.

As is known, the most common form of genetic variation is single nucleotide polymorphisms (SNPs) in human. With the development of NGS, the number of detected human genome SNPs grow rapidly. However, it is laborious and time-consuming to attain experimental knowledge about the possible disease association of the variants. Several methods have been developed for deleteriousness prediction of detected SNPs. However, the prediction capabilities of certain programs varied a lot. The correct prediction of the nonsynonymous SNVs pathogenicity of individual program may result in very poor performance. Here we developed a new method named MPPS for pathogenicity prediction of nonsynonymous SNVs. As is known, several prediction programs are unable to predict pathogenicity for specific sets of variants^29^. Figure 1 showed that the percentage of correct predictions of MPPS was higher than other methods for both pathogenic variants and neutral variants. In the first step of MPPS analysis, we found that a parameter of 3 (3 prediction methods with the most correct predictions for ‘damaging’ SNVs and 3 prediction methods with the most correct predictions for ‘benign’ SNVs) could provide the best performance for pathogenicity prediction. When evaluating the performance of MPPS, we found that a parameter of less than 3 would reduce the accuracy of MPPS because one or two programs would dominate in the analysis process. We also found that a parameter of more than 3 would reduce the accuracy of MPPS because the accuracy will be reduced by some poor-performance programs. That’s the main reason we selected 3 prediction methods for both ‘damaging’ SNVs and ‘benign’ SNVs.

After obtaining the VCF files of the patients, the variants must be annotated and interpreted. This process is tedious and time-consuming, and require highly specific expertise in the domain of the analysis, not to mention the probability of the introduction of human errors. We developed a web-based framework named AADSM for automating analysis of clinical NGS data. AADSM could provide a complete solution in assisting and enhancing Mendelian diseases diagnosis. We could analyze ~20 patients at a time using AADSM. The overall running time of AADSM is related to the variant number of the VCF file. Typically, the whole process took 8 to 20 minutes for 20 samples. The data of the patients are stored and managed over AADSM in a secure manner. Before the submission of this manuscript, AADSM (http://jiyinsou.bgidx.cn/geneM/geneDiagnoseUpload.html) is still under test, and will be online in the near future.

In conclusion, we have developed a novel single test based on targeted NGS approaches for molecular diagnosis of various Mendelian disorders. In this method, we developed a new deleteriousness prediction method for nonsynonymous SNVs, and an automating annotation and diagnosis system for Mendelian diseases. A total of 303 patients were tested using this targeted NGS approaches. And we identified 219 disease causing mutations. The results proved that this method is an informative and effective approach to make a precise diagnosis for patients with Mendelian diseases, and can greatly improve the implementation of targeted NGS in clinical practice.

## Methods

### Study participants

In the current study, a total of 303 patients were enrolled between April 2015 and June 2017 in Tongji Hospital, Fuyang People’s Hospital and Renmin Hospital of Wuhan University. Clinical information of the patients was also summarized by experienced doctor. To evaluate the accuracy and stability of this technology, 3 samples derived from YH cell line (C1, C2 and C3) and 9 probands (Table 1) with known disease causing mutations were also collected. Written informed consent was obtained from all the participants or their parents before sample collection. This study was approved by the ethics committee of BGI (BGI-IRB15083).

### Capture design, library preparation and sequencing

**Figure 4:**
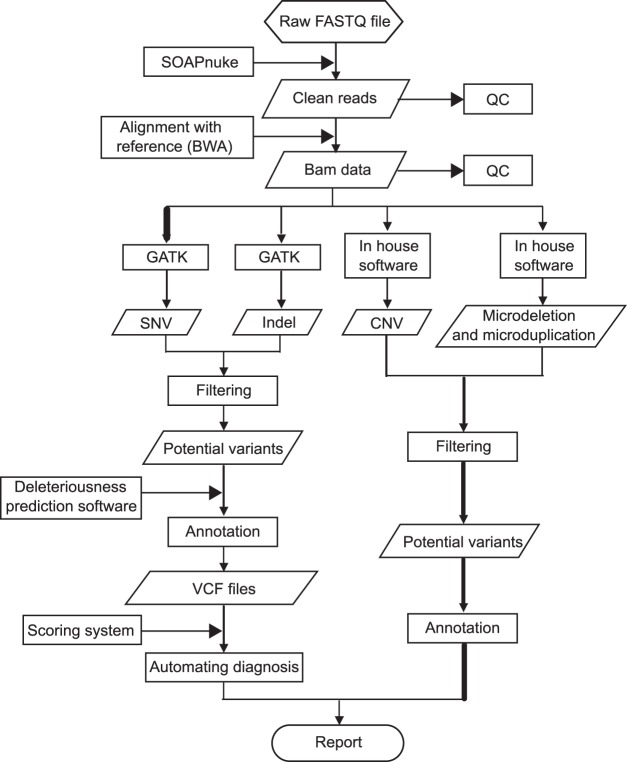
Flow diagram of bioinformatics analysis.

This study was approved by the Institutional Review Board of BGI and was carried out in accordance with the approved guidelines. We designed a new custom human array spanning 10,258,198 bp (~10 M, Roche NimbleGen). The array targets exons and 10 bp flanking intronic sequences of a total of 4,689 Mendelian diseases related genes, which were collected based on 6 well known databases (OMIM (www.omim.org), GeneReviews (www.ncbi.nlm.nih.gov/books/NBK1116), Orphanet (www.orpha.net), GENE Tests (www.genetests.org), CTGT (ctgt.net) and Genetics Home Reference (ghr.nlm.nih.gov)). A total of 4,413 Mendelian diseases can be diagnosed using this array. The 4,413 Mendelian diseases can be classified into 19 clinical categories (Fig. 4), including 870 Neuromuscular system disease, 547 Metabolic disease, 522 Growth and development abnormal, 344 Eye disease, 299 Bone disease, 241 Mental retardation and related syndromes, 238 Skin disease, 224 Circulatory system diseases, 203 Immune and infectious diseases, 173 Deafness and related syndromes, 169 Blood disease, 146 Endocrine system diseases, 111 Urinary system disease, 73 Reproductive system diseases, 72 Digestive system diseases, 51 Psychiatric and behavioral abnormal, 48 Cancer and Tumor Syndrome, 47 Respiratory system diseases, and 35 Oral disease.

Using the QIAamp DNA Blood Midi Kit (Qiagen, Hilden, Germany), genomic DNA of all the participants was isolated from the samples’ peripheral blood according to the manufacturer’s standard procedure. Library preparation was conducted at BGI following the guide of the standard protocol^1^. Briefly, 1 µg of genomic DNA was randomly fragmented to ~250 bp (Covaris S2). Next, end repair and A-tailing were performed. Then, adapters were ligated to both ends of the fragments. After purification, the adaptor-ligated DNA were subsequently amplified, purified, pooled, and hybridized to our ~10 M human array for enrichment. After library quality control, NGS libraries were then sequenced on HiSeq2500 or HiSeq4000 (Illumina) to generate paired-end reads.

### Workflow of targeted NGS approaches

**Figure 5:**
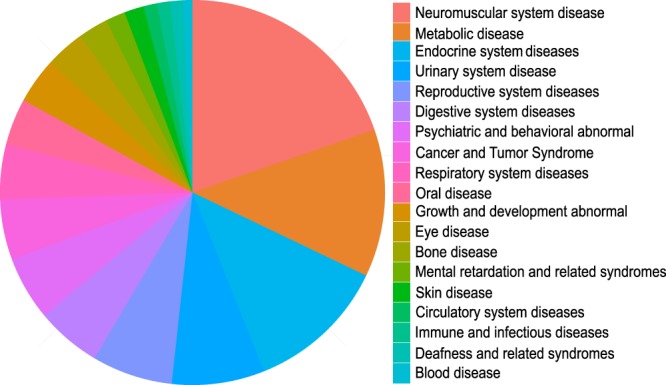
Disease classification of the 10 M chip.

In this study, we developed a new bioinformatics analysis pipeline, including data filteration, sequence alignment, variants calling, annotation and automating reporting (Fig. 5).

First, raw sequencing reads (FASTQ files) were evaluated using SOAPnuke to generate “clean reads” for further analysis (removing low quality bases, adapters and other technical sequences). After reads filtering, the 100 bp clean reads were then aligned to human reference genome (hg19) with BWA and generated sorted BAM files using Picard. To improve read mapping, we then used GATK and Picard to perform local realignment and removal of PCR duplicates. Next, we used GAKT and in-house scripts to detect sequence variants (SNVs, Indels, CNVs, and microdeletions/microduplications) as reported before^1^. Variant annotation was performed using ANNOVAR^34^ with some modifications. In the annotation step, we downloaded 5 well known databases to facilitate annotation, including HGMD (www.hgmd.org), dbSNP (www.ncbi.nlm.nih.gov/SNP), 1000 Genome (www.1000genomes.org), HapMap (www.hapmap.ncbi.nlm.nih.gov), dbNSFP (www.varianttools.sourceforge.net/Annotation/DbNSFP) and a local database of our own^11^.

For SNVs detection, we developed a new pathogenicity prediction method MPPS (Mutation Pathogenicity Prediction Software) in this study. MPPS combined a total of 17 current deleteriousness-scoring methods and mainly took 3 steps to predict the pathogenicity of nonsynonymous SNVs. In the first step, choose the best 6 prediction methods (3 prediction methods with the most correct predictions for ‘damaging’ SNVs and 3 prediction methods with the most correct predictions for ‘benign’ SNVs) according to the position of a variant. When evaluating the performance of 17 deleteriousness prediction methods, we found that the prediction capabilities of certain programs varied a lot in human genome. For example, the percentage of correct predictions rate for ‘benign’ SNVs of GERP++ is high in some region, but low in other region of the human genome. So we randomly selected subsets of SNPs in human genome to obtain the percentage of correct predictions of the 17 pathogenicity prediction methods. Then we compared the results obtained for the same region to choose the best 6 prediction methods. In the second step, calculate the prediction score of the variant according to the weight of the best prediction methods (3 methods for ‘damaging’ prediction and 3 methods for ‘benign’ prediction). In this step, we assigned the variant a score of 10 when it is classified as ‘damaging’, a score of 5 when it is classified as ‘possibly damaging’, a score of 0 when it could not be classified, a score of −5 when it is classified as ‘possibly benign’, and a score of −10 when it is classified as ‘benign’ by a prediction method. The weight of a prediction method is defined as the percentage of correct predictions in the region. The prediction score of the variant from MPPS is the sum of the scores provided by individual tools according to the weight of the best prediction methods. In the last step, evaluate the pathogenicity of the variant. First, examine whether the variant is located in conserved positions using GERP++ and phyloP100way. If the variant is located in conserved positions, the prediction score of the variant from MPPS will be added one to the original quantity. A final score of −10 (benign) to 10 (damaging) will be generated for the best 3 ‘damaging’ SNV prediction methods and the best 3 ‘benign’ SNV prediction methods respectively. If the results from the best 3 ‘damaging’ SNV prediction methods and the best 3 ‘benign’ SNV prediction methods are the same, the pathogenicity of the variant will be reported, if not, this procedure will be performed again by choosing the second best prediction methods until the results from the 3 ‘damaging’ SNV prediction methods and the 3 ‘benign’ SNV prediction methods are the same. This will be repeated until the results are the same, if not, the variant will be reported mis-classified.

We also developed an automating diagnosis and reporting system (AADSM, Automating Annotation and Diagnosis System for Mendelian disease) for clinical scientists to help themselves in assisting and enhancing diagnostics of Mendelian diseases. Finally, clinical reports of the patients will be generated.

### Validation

Clinical disease causing mutations identified using the HiSeq platform in the patients were confirmed by methods other than targeted NGS. If a pathogenic mutation was detected, segregation analysis was also performed for confirmation when the samples were available. For the validation and co-segregation analysis among the proband’s family members, we performed Sanger sequencing. We amplified regions of interest using polymerase chain reaction for sequencing (ABI 3730).

### Data Availability

The datasets generated during and/or analyzed during the current study are not publicly available, but are available from the corresponding author on reasonable request.

## Electronic supplementary material

Supplementary Material
