# Supplementary material for: Targeted next-generation sequencing as a comprehensive test for Mendelian diseases: a cohort diagnostic study
Source: Sci Rep. 2018 Aug 3;8:11646. doi: 10.1038/s41598-018-30151-z (PMC6076228; doi:10.1038/s41598-018-30151-z)
Supplement: Supplementary file 1 — Supplementary Material [file 41598_2018_30151_MOESM1_ESM.docx]

**Supplementary Material**

**Targeted next-generation sequencing as a comprehensive test for Mendelian diseases: a cohort diagnostic study**

**Authors:** Yan Sun^1,2,3†^, Jianfen Man^3†^, Yang Wan^4†^, Gao Pan^3^, Lique Du^3^, Long Li^3^, Yun Yang^3^, Liru Qiu^5^, Qing Gao^6^, Handong Dan^6^, Liangwei Mao^3^, Zhengyu Cheng^3^, Chen Fan^3^, Jing Yu^2,7^, Mufei Lin^2,7^, Karsten Kristiansen^1^, Yin Shen^6*^ and Xiaoming Wei^3*^

^1^Department of Biology, University of Copenhagen, Copenhagen, DK-2200, Denmark

^2^ BGI Genomics, BGI-Shenzhen, Shenzhen 518083, China

^3^BGI-Wuhan, BGI-Shenzhen, Wuhan, 430074, China

^4^Fuyang People’s Hospital, Fuyang, 236000, China

^5^The Nephrology Division of Department of Pediatrics, Tongji Hospital, Tongji Medical College, Huazhong University of Science and Technology, Wuhan, 430030, China

^6^Eye Center, Renmin Hospital of Wuhan University, Wuhan University, Wuhan, 430060, China

^7^China National GeneBank, BGI-Shenzhen, Shenzhen, 518120, China

* Correspondence: Yin Shen: [yinshen@whu.edu.cn](mailto:yinshen@whu.edu.cn); Xiaoming Wei: [weixm@genomics.cn](mailto:weixm@genomics.cn)

† YS, JFM and YW contributed equally to this work.

**1. Patients with positive result**

| Sample Name | Gender | Age | Mutation | Mutation Type | Mutation Status | Final Diagnosis | Mode Inheritance | Validation |
| --- | --- | --- | --- | --- | --- | --- | --- | --- |
| WHP1 | Male | 14 years | PYGM(NM_005609.2):c.1948C>T(p.R650*) | Hom | PMID:17324573 | Glycogen Storage Disease type V | AR | N.A. |
| WHP2 | Female | 33 years | GJA3(NM_021954.3):c.199G>C(p.D67H) | Het | Novel | Cataract 14 | AD | N.A. |
| WHP3 | Female | 63 years | GJA3(NM_021954.3):c.199G>C(p.D67H) | Het | Novel | Cataract 14 | AD | Yes |
| WHP4 | Female | 26 years | MIP(NM_012064.3):c.605G>A(p.W202*) | Het | PMID:23508780 | Cataract 15 | AD | N.A. |
| WHP5 | Male | 26 years | HSF4(NM_001040667.2):c.179C>T(p.P60H) | Hem | Novel | Cataract 5 | AD | N.A. |
| WHP6 | Female | - | EVC(NM_153717.2):c.1436C>T(p.P479L) | Het | Novel | Weyers Acrofacial Dysostosis | AD | N.A. |
| WHP7 | Male | 9 years | CFHR1(NM_002113.2)and CFHR3(NM_021023.5):whole genes deletion | Hom | Novel | Hemolytic Uremic syndrome susceptibility to 1 | AD | N.A. |
| WHP8 | Male | 1 years | ATP6V0A4(NM_020632.2):c.1180G>A(p.A394T)/c.620_621insC(p.L208Sfs*33 ) | Het | Novel | Autosomal Recessive Distal Renal Tubular Acidosis | AR | N.A. |
| WHP9 | Male | 1 years | BBS1(NM_024649.4):c.1772C>T(p.A591V)/c.1121C>G(p.T374S) | Het | Novel | Bardet-Biedl Syndrome 1 | AR | Yes |
| WHP10 | Female | 3 years | FCN3(NM_003665.2):c.349delC(p.L117Sfs*65)/c.498G>C(p.E166D) | Het | PMID:19535802; PMID:19501910; PMID:18289682 | Immunodeficiency due to ficolin 3 deficiency | AD | Yes |
| WHP11 | Female | 3 years | TCIRG1 (NM_006019.3):c.1555-2A>C /c.1775G>A(p.W592*) | Het | Novel | Autosomal Recessive Osteopetrosis 1 | AR | Yes |
| WHP12 | Male | 41 years | PAX6(NM_001258462.1):c.120C>A(p.C40*) | Het | PMID:21850189; PMID:16785853 | Foveal Hypoplasia and Presenile Cataract Syndrome | AD | N.A. |
| WHP13 | Female | 6 years | HSF4(NM_001040667.2):c.558C>T(p.G186G) | Hom | Novel | Cataract 5 | AD | N.A. |
| WHP14 | Male | 7 years | IFT140 (NM_014714.3)):c.1219C>T(p.R407W)/c.2446C>T(p.R816W) | Het | Novel | Short-rib thoracic dysplasia 9 with or without polydactyly | AR | Yes |
| WHP15 | Male | 9 years | COL4A4(NM_000092.4):c.1624-1G>A; TSC1(NM_000368.4):c.1460C>G(p.S487C) | Het | Novel | Autosomal Dominant Alport Syndrome;Tuberous Sclerosis 1 | AD | N.A. |
| WHP16 | Female | 27 years | MIP(NM_012064.3):c.494G>A(p.G165D) | Het | PMID:23116563 | Cataract 15 | AD | N.A. |
| WHP17 | Male | 31 years | PMP22(NM_153322.1):c.319+1G>T; MYH14(NM_001145809.1):c.3040G>C(p.E1014Q) | Het | PMID:8012365 | Charcot-Marie-Tooth disease;Peripheral Neuropathy, Myopathy, Hoarseness, And Hearing Loss | AD | N.A. |
| WHP18 | Female | 37 years | IMPDH1 (NM_000883.3):c.626C>T(p.S209L) | Het | Novel | Retinitis pigmentosa 10 | AD | N.A. |
| WHP19 | Male | 31 years | IMPDH1 (NM_000883.3):c.626C>T(p.S209L) | Het | Novel | Retinitis pigmentosa 10 | AD | N.A. |
| WHP20 | Male | 49 years | VCAN (NM_004385.4):c.7870G>A(p.E2624K) | Het | Novel | VCAN-Related Vitreoretinopathy | AD | N.A. |
| WHP21 | Male | 67 years | LMNB1(NM_005573.3):c.1365C>T(p.R455R) | Het | Novel | Adult-Onset Leukodystrophy | AD | N.A. |
| WHP22 | Male | 89 days | SLC2A10(NM_030777.3):c.685C>T(p.R229*)/c.752T>C(p.L251P) | Het | PMID:19781076 | Arterial tortuosity syndrome | AR | Yes |
| WHP23 | Male | 11 years | GJB1(NM_000166.5):c.8G>A(p.W3*) | Hem | Novel | X-linked Charcot-Marie-Tooth disease 1 | XL | Yes |
| WHP24 | Female | 6 months | ATP6V1B1 (NM_001692.3):c.368-1G>A/c.1354delT(p.F452Lfs*35) | Het | Novel | Distal Renal Tubular Acidosis with Progressive Nerve Deafness | AR | N.A. |
| WHP25 | Female | - | GUSB(NM_000181.3):c.1192C>T(p.R398C) | Hom | Novel | Mucopolysaccharidosis type VII | AR | N.A. |
| WHP26 | Male | 41 years | RHO (NM_000539.3):c.403C>T(p.R135W) | Het | Novel | Retinitis pigmentosa 4 | AD | N.A. |
| WHP27 | Female | 36 years | RP1 (NM_006269.1):c.1437G>T(p.M479I) | Het | PMID:12048676 | Retinitis pigmentosa 1 | AD | N.A. |
| WHP28 | Female | 60 years | USH2A (NM_206933.2):c.13465G>A(p.G4489S)/c.8641_8642insTATT(p.S2881Lfs*9) | Het | Novel | Retinitis pigmentosa 39 | AR | N.A. |
| WHP29 | Female | 40 years | USH2A (NM_206933.2):c.9958G>T(p.G3320C)/c.99_100insT(p.R34Sfs*41) | Het | PMID:25133613; PMID:23661369 | Retinitis pigmentosa 39 | AR | N.A. |
| WHP30 | Male | 5 years | OCRL(NM_000276.3):c.1040G>A(p.G347E); SLC7A9(NM_001243036.1):c.829G>A(p.V277M) | Hem; Het | Novel | Dent Disease 2; Cystinuria | AD | N.A. |
| WHP31 | Male | 3 years | SLC12A3(NM_000339.2):c.1362_1363insC(p.L457Dfs*68)/c.2029G>A(p.V677M) | Het | Novel | Gitelman syndrome | AR | N.A. |
| WHP32 | Female | 25 years | ARHGEF10 (NM_014629.2):c.824G>A(p.R275H); PMP22(NM_153322.1):whole gene duplication | Het | Novel | Slowed Nerve Conduction Velocity;Charcot-Marie-Tooth disease | AD | N.A. |
| WHP33 | Male | 7 years | EPHA2 (NM_004431.3):c.944G>A(p.R315Q); MYOC (NM_000261.1):c.1432G>T(p.D478Y) | Het | Novel | Cataract 6;Primary Open Angle Glaucoma 1A | AD | N.A. |
| WHP34 | Female | 2 years | NLRP3 (NM_004895.4):c.214G>A(p.V72M) | Het | Novel | Muckle-Wells syndrome | AD | N.A. |
| WHP35 | Male | 4 years | ADCK4 (NM_024876.3):c.748G>C(p.D250H)/c.737G>A(p.S246N) | Het | Novel | Nephrotic Syndrome Type 9 | AR | Yes |
| WHP36 | Male | 12 years | CLCN5 (NM_001127899.1):c.992_993insAGTATTAT(p.F334Xfs*1) | Hem | Novel | Dent Disease 1 | XL | Yes |
| WHP37 | Male | - | GJA8(NM_005267.4):c.154T>C(p.F52L) | Het | Novel | Cataract 1 | AD | N.A. |
| WHP38 | Female | - | GJA3(NM_021954.3):c.1152_1153insG(p.S385Efs*83) | Het | Novel | Cataract 14 | AD | N.A. |
| WHP39 | Female | 23 years | CRYBB2(NM_000496.2):c.152C>T(p.S51F) | Het | Novel | Cataract 3 | AD | N.A. |
| WHP40 | Male | 18 years | ATP7B(NM_000053.3):c.2333G>T(p.R778L)/c.2310C>G(p.L770L) | Het | PMID:21796144; PMID:18034201 | Wilson Disease | AR | N.A. |
| WHP41 | Male | 28 years | MPZ (NM_000530.6):c.286A>C(p.K96Q);DYNC1H1(NM_001376.4):c.12804C>T(p.F4268F) | Het | Novel | Charcot-Marie-Tooth disease;Autosomal Dominant Lower Extremity-Predominant Spinal Muscular Atrophy 1 | AD | N.A. |
| WHP42 | Male | 6 years | CFHR5(NM_030787.3):c.508G>A(p.V170M) | Het | Novel | Nephropathy due to CFHR5 deficiency | AD | N.A. |
| WHP43 | Male | 11 years | COL4A4(NM_000092.4):c.4333G>A(p.G1445R)/c.1505delC(p.P502Lfs*151) | Het | Novel | Alport Syndrome | AD | Yes |
| WHP44 | Male |  | CYP4V2 (NM_207352.3):c.802-6_810delATACAGGTCATCGCT | Hom | Novel | Bietti Crystalline Dystrophy | AR | N.A. |
| WHP45 | Male | 7 years | COL4A5(NM_033380.2):c.3817G>T(p.G1273C) | Hem | Novel | X-linked Alport Syndrome | XL | Yes |
| WHP46 | Female | 5 years | COL4A5(NM_033380.2):c.901G>C(p.G301R) | Het | Novel | X-linked Alport Syndrome | XL | Yes |
| WHP47 | Male | 28 years | MFN2(NM_014874.3):c.385A>G(p.T129A) | Het | Novel | Charcot-Marie-Tooth disease type 2A2 | AD | N.A. |
| WHP48 | Male | 26 years | CRYBB2 (NM_000496.2):c.107_115delGGCCCTGCCinsCGAGTTTCCAACCTGAAGTTT | Het | Novel | Cataract 3 | AD | Yes |
| WHP49 | Male | 22 years | MYOC(NM_000261.1):c.136C>T(p.R46*) | Het | Novel | Primary Open Angle Glaucoma 1A | AD | N.A. |
| WHP50 | Female | 37 years | EXT1 (NM_000127.2):c.957T>G(p.Y319*) | Het | Novel | Hereditary Multiple Osteochondromatosis Type I | AD | N.A. |
| WHP51 | Male | 2 years | ANLN(NM_018685.2):c.3062A>T (p.D1021V) | Het | Novel | Focal Segmental Glomerulosclerosis 8 | AD | Yes |
| WHP52 | Male | 29 years | GJA8(NM_005267.4):c.134G>C(p.W45S) | Het | PMID:18334946 | Cataract 1 | AD | N.A. |
| WHP53 | Male | 30 years | CRYBB2(NM_000496.2):c.487C>T(p.Q163*) | Het | Novel | Cataract 3 | AD | Yes |
| WHP54 | Female | 23 years | BFSP2(NM_003571.2):c.113G>A(p.S38N) | Het | Novel | Cataract 12 | AD | Yes |
| WHP55 | Female | 11 years | CRYBB2(NM_000496.2):c.463C>T(p.Q155*) | Het | PMID:19321936 | Cataract 3 | AD | N.A. |
| WHP56 | Female | 28 years | COL4A4 (NM_000092.4):c.4214_4215insC(p.G1406Rfs*27) | Het | Novel | Alport Syndrome | AD/AR | Yes |
| WHP57 | Male | 1 years | INF2 (NM_022489.3):c.1978C>T(p.R660W); COL4A4 (NM_000092.4):c.3647G>C(p.G1216A); CFH(NM_000186.3):c.3566T>G(p.L1189R) | Het | Novel | Focal Segmental Glomerulosclerosis 5; Alport Syndrome; Hemolytic Uremic syndrome susceptibility to 1 | AD | Yes |
| WHP58 | Female | 22 years | COL4A5(NM_033380.2):c.232-6_244delTTTCAGGGTGATGATGGAA | Het | Novel | X-linked Alport Syndrome | XL | Yes |
| WHP59 | Male | 4 years | NTRK1(NM_002529.3):c.632T>A(p.V211E)/c.1253_1254delTC(p.S419Gfs*80) | Het | Novel | Hereditary Sensory and Autonomic Neuropathy IV | AR | N.A. |
| WHP60 | Female | - | TDRD7(NM_014290.2):c.688_689insA(p.Y230Terfs*1); CRYBA2(NM_057094.1):c.457T>C(p.Y153H) | Het | Novel | Cataract 36;Cataract 42 | AD | N.A. |
| WHP61 | Male | 3 years | OCRL(NM_000276.3):CDS20-23 deletion | Hem | Novel | Lowe Syndrome | XL | N.A. |
| WHP62 | Male | 36 years | AGXT(NM_000030.2):c.332G>A (p.R111Q)/c.815_816insGA(p.S275Rfs*38) | Het | Novel | Primary Hyperoxaluria Type 1 | AR | N.A. |
| WHP63 | Female | 23 years | PAX6(NM_001258462.1):c.817_818insT(p.S273Ffs*2) | Het | Novel | Aniridia 1 | AD | N.A. |
| WHP64 | Female | 27 years | FBN2(NM_001999.3):c.4217G>A(p.C1406Y); MYH8( NM_002472.2):c.4288G>T(p.D1430Y) | Het | Novel | Congenital Contractural Arachnodactyly;Distal Arthrogryposis type 7 | AD | N.A. |
| WHP65 | Male | 2 years | CLCN5 (NM_001127899.1):c.1272delG(p.K424Nfs*5) | Hem | Novel | Dent Disease 1 | XL | N.A. |
| WHP66 | Female | 11 years | BBS2 (NM_031885.3):c.943C>T(p.R315W)/c.534+1G>T | Het | PMID:11567139; PMID:24280758 | Bardet-Biedl Syndrome 2 | AR | N.A. |
| WHP67 | - | - | BBS2(NM_031885.3)c.647G>C (p.R216P)/c.534+1G>T | Het | PMID:24280758 | Bardet-Biedl Syndrome 2 | AD | Yes |
| WHP68 | Female | 27 years | CNGA1(NM_001142564.1):c.829G>A (p.D277N)/c.472delC(p.L158Ffs*4) | Het | Novel | Retinitis pigmentosa 49 | AR | Yes |
| WHP69 | Male | - | IGHMBP2 (NM_002180.2): c.344C>T(p.T115M)/CDS10-14 duplication | Het | PMID:26136520;PMID:26922252 | Autosomal Recessive Distal Spinal Muscular Atrophy 1;Charcot-Marie-Tooth disease | AR | N.A. |
| WHP70 | Female | 22 years | EPHA2(NM_004431.3):c.983C>A(p.P328H) | Het | Novel | Cataract 6 | AD | N.A. |
| WHP71 | Male | 5 years | XPA(NM_000380.3):c.283G>A(p.G95R)/c.631C>T(p.R211*) | Het | PMID:1372103 | Xeroderma Pigmentosum Group A | AR | N.A. |
| WHP72 | Female | 29 years | RP1L1(NM_178857.5):c.32C>T(p.P11L) | Het | Novel | Occult Macular Dystrophy | AD | N.A. |
| WHP73 | Male | 26 years | GJA8(NM_005267.4):c.163A>G(p.N55D) | Het | Novel | Cataract 1 | AD | N.A. |
| WHP74 | Male | 11 years | IQCB1(NM_001023570.2):c.1090C>T(p.R364*)/c.1225C>T(p.Q409*) | Het | Novel | Senior-Loken syndrome 5 | AR | Yes |
| WHP75 | Male | 35 years | USH2A(NM_206933.2):c.4165delG(p.V1389Lfs*43)/c.11156G>A(p.R3719H) | Het | PMID:25133613;PMID:26496393;PMID:25649381 | Retinitis pigmentosa 39 | AR | N.A. |
| WHP76 | Female | 26 years | CRYBB3(NM_004076.3):c.593G>A(p.R198H) | Het | Novel | Cataract 22 | AD | N.A. |
| WHP77 | Male | 21 years | SLC12A3(NM_000339.2):c.468C>A(p.Y156*) | Hom | Novel | Gitelman syndrome | AR | N.A. |
| WHP78 | Female | 39 years | FLG (NM_002016.1):c.5841G>A(p.W1947*)/c.3321delA(p.G1109Efs*13) | Het | PMID:25997159; PMID:18841000;PMID:17291859;PMID:19958351 | Ichthyosis Vulgaris | AD | N.A. |
| WHP79 | Female | 9 years | FLG (NM_002016.1):c.3321delA(p.G1109Efs*13) | Het | PMID:18841000;PMID:17291859;PMID:19958351 | Ichthyosis Vulgaris | AD | N.A. |
| WHP80 | Male | 42 years | CRYBB3(NM_004076.3):c.466G>A(p.G156R) | Het | PMID:27307692 | Cataract 22 | AD | N.A. |
| WHP81 | Female | 8 years | WT1(NM_024426.4) :c.1432+5G>A | Het | PMID:15973330;PMID:14646409 ;PMID:27300205 | Denys-Drash syndrome; Meacham Syndrome; Frasier syndrome | AD | Yes |
| WHP82 | Female | 27 years | LONP1(NM_004793.2):c.2392G>A(p.G798S)/c.2290G>A(p.V764M) | Het | Novel | CODAS Syndrome | AR | Yes |
| WHP83 | Female | 35 years | FLG(NM_002016.1):c.3905C>A(p.S1302*) | Het | Novel | Ichthyosis Vulgaris | AD | N.A. |
| WHP84 | Female | 62 years | FLG(NM_002016.1):c.3905C>A(p.S1302*) | Het | Novel | Ichthyosis Vulgaris | AD | N.A. |
| WHP85 | Female | 8 years | FLG(NM_002016.1):c.3905C>A(p.S1302*) | Het | Novel | Ichthyosis Vulgaris | AD | N.A. |
| WHP86 | Female | 3 years | NHS(NM_198270.2):c.985G>T(p.E329*) | Het | Novel | Cataract 40 | AD | N.A. |
| WHP87 | Female | 29 years | CFB(NM_001710.5):c.1598 A>G(p.K533R) | Het | PMID:20108004;PMID:27064621 ;PMID:20513133 | Hemolytic Uremic syndrome susceptibility to 4 | AD | N.A. |
| WHP88 | Female | 18 years | COL4A5(NM_033380.2):c.2215C>G(p.P739A) | Hem | PMID:10684360 | X-linked Alport Syndrome | XL | N.A. |
| WHP89 | Female | 48 years | GJA8(NM_005267.4):c.773C>T(p.S258F) | Het | PMID:20597646 ;PMID:25301372 | Cataract 1 | AD | N.A. |
| WHP90 | - | - | ALDH7A1(NM_001182.4):c.1279G>C(p.E427Q)/CDS8-13 deletion | Het | PMID:22371912 ;PMID:23430810 ;PMID:26995068 | Pyridoxine-Dependent Epilepsy | AR | N.A. |
| WHP91 | Female | 3 years | SCN9A(NM_002977.3):c.4174-1G>C | Het | Novel | Congenital Insensitivity to Pain | AD/AR | N.A. |
| WHP92 | Female | 7 years | SCN9A(NM_002977.3):c.850delG(p.E284Kfs*3)/c.129_141delTGAAGAAGCCCCA(p.D43Efs*43) | Het | Novel | Congenital Insensitivity to Pain | AD/AR | N.A. |
| WHP93 | Female | 2 years | SCN9A(NM_002977.3):c.296G>A(p.R99H)/c.2749T>G(p.W917G) | Het | Novel | Congenital Insensitivity to Pain | AD/AR | N.A. |
| WHP94 | Male | 8 years | SCN9A(NM_002977.3):c.2697G>A(p.M899I) | Het | PMID:21939494 | Congenital Insensitivity to Pain | AD/AR | N.A. |
| WHP95 | Male | 15 years | CBS (NM_001178009.1):c.526G>A(p.E176K)/c.949A>G(p.R317G) | Het | PMID:20308073 ; PMID:11359213 | Homocystinuria Caused by Cystathionine Beta-Synthase Deficiency | AR | Yes |
| WHP96 | Male | 1 years | CFH (NM_000186.3):c.3643C>G(p.R1215G); CD46(NM_172359.2):c.1114A>T(p.R372W) | Het | PMID:9551389; PMID:25261570 | Hemolytic Uremic syndrome susceptibility to 1;Hemolytic Uremic syndrome susceptibility to 2 | AD | Yes |
| WHP97 | Male | 26 years | RHBDF2(NM_024599.5):c.566C>T(p.P189L) | Het | PMID:22265016 | Tylosis with Esophageal Cancer | AD | N.A. |
| WHP98 | Male | 4 months | AVPR2(NM_000054.4)whole gene deletion | Hem | PMID:11754100; PMID:8037205; PMID:22879391 | X-Linked Nephrogenic Diabetes Insipidus | XL | N.A. |
| WHP99 | Male | 8 years | IFT172(NM_015662.1):c.2053C>T(p.R685*)/c.5071T>C(p.Y1691H) | Het | Novel | Short-rib thoracic dysplasia 10 with or without polydactyly | AR | N.A. |
| WHP100 | Female | 12 years | GSN(NM_000177.4):c.444G>T(p.E148D); COL4A4(NM_000092.4):c.930+1G>A; TNXB(NM_019105.6):c.8201_8202insC(p.E2735Terfs*1) | Het | Novel | Finnish type Amyloidosis;Alport Syndrome;Vesicoureteral Reflux 8 | AD/AR | N.A. |
| WHP101 | Female | 9 years | TSC2(NM_000548.3):c.977delC(p.M327Wfs*36) | Het | Novel | Tuberous Sclerosis 2 | AD | Yes |
| WHP102 | Male | 53 years | EYS(NM_001142800.1):c.8545C>T (p.R2849*)/c.5644+5G>A | Het | Novel | Retinitis pigmentosa 25 | AR | N.A. |
| WHP103 | Male | 46 years | ABCA4(NM_000350.2):c.4720G>T(p.E1574*)/c.6289C>T(p.P2097S) | Het | PMID:19365591 | Age-Related Macular Degeneration 2;Stargardt Disease 1 | AD/AR | N.A. |
| WHP104 | Male | 47 years | USH2A(NM_206933.2):c.142_143insGA(p.K48Rfs*98)/c.2802T>G(p.C934W) | Het | PMID:21686329 ; PMID:25649381; PMID:25078356 | Retinitis pigmentosa 39 | AR | N.A. |
| WHP105 | Male | 13 years | CFB (NM_001710.5):c.1697A>C (p.E566A) | Het | PMID:23847193 ; PMID:25400666 | Hemolytic Uremic syndrome susceptibility to 4 | AD | N.A. |
| WHP106 | Male | 32 years | FBN1(NM_000138.4):c.385T>C(p.C129R) | Het | PMID:22539340; PMID:21907952 | Marfan Syndrome | AD | N.A. |
| WHP107 | Male | 5 years | ZNF423(NM_015069.2):c.1801G>A (p.A601T)/c.1537G>A(p.G513S) | Het | Novel | Nephronophthisis 14 | AR | N.A. |
| WHP108 | Male | 6 years | AGXT (NM_000030.2):c.557C>T (p.A186V)/c.590G>A(p.R197Q); OCRL(NM_000276.3):c.808G>A(p.D270N) | Het/Hem | PMID:19479957; PMID:19245173 | Primary Hyperoxaluria Type 1;Dent Disease 2 | AR/XL | N.A. |
| WHP109 | Female | 51 years | NOTCH3(NM_000435.2):c.505C>T(R169C) | Het | PMID:25982499;PMID:19174371 | Cerebral Autosomal Dominant Arteriopathy with Subcortical Infarcts And Leukoencephalopathy Type 1 | AD | Yes |
| WHP110 | Male | 4 years | PPT1(NM_000310.3):c.722C>T(p.S241L)/CDS9 deletion | Het | PMID:22387303 | Neuronal Ceroid-Lipofuscinoses 1 | AR | Yes |
| WHP111 | Male | 4 years | MMACHC(NM_015506.2):c.82-2A>G/c.463G>C(p.G155R) | Het | PMID:26149271; PMID:23837176 | Methylmalonic aciduria and homocystinuria CblC type | AR | Yes |
| WHP112 | Male | 50 years | PKD1(NM_001009944.2):c.7288C>T(p.R2430*) | Het | PMID:22508176; PMID:22185115 ; PMID:11012875 | Polycystic kidney disease 1 | AD | N.A. |
| WHP113 | Male | 21 years | PMP22(NM_153322.1):Whole gene duplication | Het | PMID:17620487; PMID:14627690 | Hereditary Neuropathy With Liability To Pressure Palsies | AD | N.A. |
| WHP114 | Male | 35 years | FBN1 (NM_000138.4):c.5839T>C(p.C1947R) | Het | Novel | Marfan Syndrome | AD | N.A. |
| WHP115 | Female | 7 years | COL4A5(NM_033380.2):c.262C>T(p.P88S) | Hem | PMID:22921432 | X-linked Alport Syndrome | XL | Yes |
| WHP116 | Male | 52 years | PMP22(NM_153322.1):Whole gene duplication |  | PMID:17620487; PMID:14627690 | Hereditary Neuropathy With Liability To Pressure Palsies | AD | N.A. |
| WHP117 | Female | 8 years | PKHD1(NM_138694.3):c.9455delA (p.N3152Tfs*10)/c.5231A>G(p.N1744S) | Het | Novel | Autosomal Recessive Polycystic Kidney Disease | AR | Yes |
| WHP118 | Male | 6 years | TTC21B(NM_024753.4):c.1552T>C(p.C518R)/c.895T>C(p.C299R) | Het | Novel | Nephronophthisis 12 | AR | Yes |
| WHP119 | Male | 37 years | PMP22(NM_153322.1):Whole gene duplication | Het | PMID:1674726 ; PMID:1303228; PMID:1303282 | Charcot-Marie-Tooth disease | AD | Yes |
| WHP120 | Female | 40 years | CLCN1(NM_000083.2):c.847C>T(p.L283F) | Het | PMID:12390967 | Myotonia Congenita | AD | N.A. |
| WHP121 | Male | 48 years | DCTN1(NM_004082.4):c.1588G>A(p.V530M) | Het | Novel | Distal Hereditary Motor Neuronopathy type VIIB | AD | N.A. |
| WHP122 | Male | 2 months | AVPR2(NM_000054.4):whole gene deletion | Het | PMID:11754100 ; PMID:8037205 ; PMID:22879391 | X-Linked Nephrogenic Diabetes Insipidus | XL | N.A. |
| WHP123 | Female | 4 years | COL4A3(NM_000091.4):c.1106G>A(p.G369D) | Het | Novel | Alport Syndrome; Benign Familial Hematuria | AD/AR | Yes |
| WHP124 | Male | 7 years | CFHR1(NM_002113.2):CDS2-6 deletion;CFHR3(NM_021023.5):Whole gene duplication | Hom | PMID:19531976; PMID:23243267 ; PMID:26163426 | Hemolytic Uremic syndrome susceptibility to 1 | AD | Yes |
| WHP125 | Male | 8 years | SLC12A3 (NM_000339.2):c.1856G>A (p.G619D)/c.2451_2458delCCCCAAGG(p.K819Gfs*26) | Het | PMID:27454426; | Gitelman syndrome | AR | Yes |
| WHP126 | Male | 10 years | COL4A5(NM_033380.2):c.5038C>T(p.R1680*) | Hem | PMID:12105244 | X-linked Alport Syndrome | XL | Yes |
| WHP127 | Female | 14 years | NOTCH3(NM_000435.2):c.472G>A(p.D158N) | Het | Novel | Cerebral Autosomal Dominant Arteriopathy with Subcortical Infarcts And Leukoencephalopathy Type 1 | AD | Yes |
| WHP128 | Male | 2 years | MKS1(NM_017777.3): c.1601G>A(p.R534Q)/c.323G>A(p.R108H) | Het | PMID:24608809 | Joubert Syndrome 28;Bardet-Biedl Syndrome 13 | AR | Yes |
| WHP129 | Male | 9 years | PAX2(NM_003990.3):c.418C>T( p.R140W ) | Het | Novel | Focal Segmental Glomerulosclerosis 7 | AD | Yes |
| WHP130 | Female | 20 years | MPZ(NM_000530.6):c.308G>C( p.G103A ) | Het | PMID:11445635 | Charcot-Marie-Tooth disease | AD | N.A. |
| WHP131 | Male | 50 years | NOTCH3(NM_000435.2):c.1010A>G(p.Y337C) | Het | PMID:15364702 | Cerebral Autosomal Dominant Arteriopathy with Subcortical Infarcts And Leukoencephalopathy Type 1 | AD | Yes |
| WHP132 | Female | 2 years | TTC21B(NM_024753.4):c.1897C>T(p.Q633* )/c.1552T>C(p.C518R ) | Het | PMID:28124483 | Nephronophthisis 12 | AR | Yes |
| WHP133 | Male | 7 days | IMPAD1(NM_017813.4):c.700G>T (p.E234*)/CDS4-5 deletion | Het | Novel | Chondrodysplasia with Joint Dislocations | AR | Yes |
| WHP134 | Male | 13 years | COL4A5(NM_033380.2):c.465+5G>T | Hem | Novel | X-linked Alport Syndrome | XL | Yes |
| WHP135 | Male | 2 years | COL4A4(NM_000092.4):c.2311G>A(p.G771R) | Het | Novel | Alport Syndrome;Benign Familial Hematuria | AD/AR | Yes |
| WHP136 | Male | 10 years | COL4A4(NM_000092.4):c.4333+2T>C | Het | Novel | Alport Syndrome;Benign Familial Hematuria | AD/AR | N.A. |
| WHP137 | Male | 5 years | AVPR2(NM_000054.4):c.409C>T(p.R137C) | Hem | Novel | Nephrogenic Syndrome of Inappropriate Antidiuresis | XL | Yes |
| WHP138 | Female | 4 months | SLC26A3(NM_000111.2):c.386C>T(p.P129L )/c.270_271insAA(p.G91Kfs*3 ) | Het | PMID:21394828; PMID:24350656; PMID:9718329 | Familial Chloride Diarrhea | AR | Yes |
| WHP139 | Male | 9 years | COL4A3(NM_000091.4):c.4664C>T( p.A1555V); COL4A4(NM_000092.4):c.870G>T(p.K290N ) | Het | Novel | Alport Syndrome;Benign Familial Hematuria | AD/AR | Yes |
| WHP140 | Female | 11 years | AGXT(NM_000030.2):c.1015delG(p.V339Sfs*2) | Hom | Novel | Primary Hyperoxaluria Type 1 | AR | Yes |
| WHP141 | Female | 5 years | SLC12A1(NM_000338.2):c.463G>A(p.G155S)/c.3207G>T(p.L1069F) | Het | Novel | Bartter Syndrome 1 | AR | Yes |
| WHP142 | Female | 11 years | SLC3A1(NM_000341.3):c.1332+2T>A | Het | Novel | Cystinuria | AD/AR | N.A. |
| WHP143 | Male | 56 years | SETX(NM_015046.5):c.7114G>A(p.D2372N) | Het | PMID:25382069 | Amyotrophic Lateral Sclerosis 4 | AD | N.A. |
| WHP144 | Male | 51 years | CHM(NM_000390.2):c.1456_1457insG(p.A486Gfs*6 ); GUCY2D(NM_000180.3):c.2375C>T(p.P792L ) | Het | Novel | Choroideremia;Cone-Rod Dystrophy 6 | AD | N.A. |
| WHP145 | Male | 2 years | NTRK1(NM_002529.3):c.851-33T>A/c.1805G>A(p.R602Q) | Het | Novel | Hereditary Sensory and Autonomic Neuropathy IV | AR | N.A. |
| WHP146 | Male | 14 years | COL4A5(NM_033380.2): c.3125G>T(p.G1042V) | Hem | Novel | X-linked Alport Syndrome | XL | Yes |
| WHP147 | Female | 30 years | SLC7A9(NM_014270.4):c.829G>A(p.V277M);INF2(NM_022489.3):c.1372C>T(p.P458S) | Het | Novel | Cystinuria;Focal Segmental Glomerulosclerosis 5 | AD | N.A. |
| WHP148 | Male | 27 years | GAA(NM_000152.3):c.953T>A(p.M318K)/c.2184delC(p.L729Wfs*35) | Het | Novel | Glycogen storage disease II | AR | Yes |
| WHP149 | Female | 45 years | PMP22(NM_000304.2):CDS2-4 duplication | Het | PMID:1674726;PMID:1303228 ;PMID:1303282;PMID:14627690 | Charcot-Marie-Tooth disease | AD | N.A. |
| WHP150 | Male | 9 years | LPIN1(NM_145693.2):c.357_358insCT(p.K121Terfs*1) | Hom | Novel | Acute Recurrent Myoglobinuria | AR | Yes |
| WHP151 | Male | 2 months | FANCI(NM_001113378.1):c.286G>A(p.E96K)/c.3457C>G(p.L1153V) | Het | Novel | Fanconi anemia | AR | Yes |
| WHP152 | Male | 3 years | RS1(NM_000330.3):c.214G>A(p.E72K) | Hem | PMID:9618178 | X-Linked Juvenile Retinoschisis | XL | Yes |

**2. Patients with negative result**

| **Sample Name** | **Gender** | **Age** | **Clinical Diagnosis According to Patient's Phenotype** | **Mutation** |
| --- | --- | --- | --- | --- |
| WHN1 | Female | 5 years | Cerebral Cavernous Malformation | Negative |
| WHN2 | Male | 59 years | Polycystic kidney and liver | Negative |
| WHN3 | Female | 3 months | Steroid-Resistant Nephrotic Syndrome | Negative |
| WHN4 | Female | - | Angle Glaucoma | Negative |
| WHN5 | Male | - | Pentalogy of Cantrell | Negative |
| WHN6 | Male | 6 years | Hemolytic Uremic syndrome | Negative |
| WHN7 | Male | 4 years | Hemolytic Uremic syndrome | Negative |
| WHN8 | Male | - | Congenital Adrenal Hyperplasia due to 21-Hydroxylase-Deficiency | Negative |
| WHN9 | Male | 3 years | Medullary cystic kidney disease | Negative |
| WHN10 | Male | 12 years | Hemolytic Uremic syndrome | Negative |
| WHN11 | Male | 8 months | Hemolytic Uremic syndrome | Negative |
| WHN12 | Male | 4 years | Angle Glaucoma | Negative |
| WHN13 | Male | 33 years | Angle Glaucoma | Negative |
| WHN14 | Female | 35 years | Retinitis pigmentosa | Negative |
| WHN15 | Female | 67 years | Spinocerebellar Ataxia | Negative |
| WHN16 | Female | 21 years | Angle Glaucoma | Negative |
| WHN17 | Male | 9 years | Familial Exudative Vitreoretinopathy | Negative |
| WHN18 | Male | 13 years | Familial Exudative Vitreoretinopathy | Negative |
| WHN19 | Female | 9 months | Hemolytic Uremic syndrome/Congenital Thrombotic thrombocytopenic purpura | Negative |
| WHN20 | Male | 23 years | Angle Glaucoma | Negative |
| WHN21 | Male | 61 years | Retinitis pigmentosa | Negative |
| WHN22 | Female | - | Cataract | Negative |
| WHN23 | Male | 27 years | Cataract | Negative |
| WHN24 | Female | 19 years | Angle Glaucoma | Negative |
| WHN25 | Male | - | Nephropathy | Negative |
| WHN26 | Male | 31 years | - | Negative |
| WHN27 | Female | 28 years | - | Negative |
| WHN28 | Male | 31 years | Cataract | Negative |
| WHN29 | Female | 8 years | Angle Glaucoma | Negative |
| WHN30 | - | - | Fetalis hydrops | Negative |
| WHN31 | - | - | Fetalis hydrops | Negative |
| WHN32 | Male | 36 years | Eye disease | Negative |
| WHN33 | Male | 28 years | Charcot-Marie-Tooth disease | Negative |
| WHN34 | Female | 37 years | Hereditary ataxia | Negative |
| WHN35 | Male | 27 years | - | Negative |
| WHN36 | Female | 12 years | Renal Tubular disease | Negative |
| WHN37 | Female | 8 years | Renal Tubular disease | Negative |
| WHN38 | Female | - | Cataract | Negative |
| WHN39 | Female | - | Cataract | Negative |
| WHN40 | Male | 26 years | Hereditary peripheral neuropathy | Negative |
| WHN41 | Female | 39 years | Hereditary peripheral neuropathy | Negative |
| WHN42 | Female | 33 years | Cataract | Negative |
| WHN43 | Male | 25 years | Hereditary peripheral neuropathy | Negative |
| WHN44 | Female | 14 years | Lysosomal disease | Negative |
| WHN45 | Female | 4 years | Citrullinemia/Tyrosinemia | Negative |
| WHN46 | Male | 12 years | Renal Tubular disease | Negative |
| WHN47 | Male | 9 years | Glomerulosclerosis disease | Negative |
| WHN48 | Male | 2 years | Renal Tubular disease | Negative |
| WHN49 | Female | 65 years | Macular Degeneration | Negative |
| WHN50 | Female | 19 years | Hereditary peripheral neuropathy | Negative |
| WHN51 | Female | - | - | Negative |
| WHN52 | Female | 9 months | Glomerulosclerosis disease | Negative |
| WHN53 | Female | 66 years | Hypokalemia disease | Negative |
| WHN54 | Female | 3 years | - | Negative |
| WHN55 | Female | 13 years | Hereditary peripheral neuropathy | Negative |
| WHN56 | Female | 2 years | Renal Tubular disease | Negative |
| WHN57 | Male | 43 years | Thyroid Hormone Resistance | Negative |
| WHN58 | Male | 7 years | - | Negative |
| WHN59 | Male | 26 years | Familial Exudative Vitreoretinopathy | Negative |
| WHN60 | Male | 8 years | Nephropathy | Negative |
| WHN61 | Male | 5 years | Familial Exudative Vitreoretinopathy | Negative |
| WHN62 | Male | 12 years | Cataract | Negative |
| WHN63 | Male | 13 years | Glomerulosclerosis disease | Negative |
| WHN64 | Male | - | Cataract | Negative |
| WHN65 | Female | 44 years | Angle Glaucoma | Negative |
| WHN66 | Female | - | Hereditary ataxia | Negative |
| WHN67 | Female | 57 years | - | Negative |
| WHN68 | Male | - | Angle Glaucoma | Negative |
| WHN69 | Male | 32 years | 3-Methylglutaconic Aciduria | Negative |
| WHN70 | Male | 35 years | Angle Glaucoma | Negative |
| WHN71 | Male | 33 years | Cataract | Negative |
| WHN72 | Male | 32 years | Cataract | Negative |
| WHN73 | Male | 5 years | Cataract | Negative |
| WHN74 | Male | 7 months | Osteogenesis Imperfecta | Negative |
| WHN75 | Male | 5 years | Cataract | Negative |
| WHN76 | Female | 26 years | Nephropathy | Negative |
| WHN77 | Male | 14 years | Pseudopseudohypoparathyroidism | Negative |
| WHN78 | Male | 11 years | Renal Tubular disease | Negative |
| WHN79 | Male | - | - | Negative |
| WHN80 | Female | 21 years | Glomerulosclerosis disease | Negative |
| WHN81 | Male | 82 years | Polycystic kidney and liver | Negative |
| WHN82 | Female | 13 years | Renal Tubular disease | Negative |
| WHN83 | Male | 4 years | Renal Tubular disease | Negative |
| WHN84 | Female | 6 years | Glomerulosclerosis disease | Negative |
| WHN85 | Male | 6 years | Renal Tubular disease | Negative |
| WHN86 | Male | 31 years | Steroid-Resistant Nephrotic Syndrome | Negative |
| WHN87 | Male | 2 years | Renal Tubular disease | Negative |
| WHN88 | - | - | Jaundice | Negative |
| WHN89 | Male | - | Hereditary muscular disease | Negative |
| WHN90 | Male | 21 years | Hereditary muscular disease | Negative |
| WHN91 | Male | 65 years | Retinitis pigmentosa | Negative |
| WHN92 | Female | 4 years | Renal Tubular disease | Negative |
| WHN93 | Female | 8 years | Nephropathy/Moyamoya disease | Negative |
| WHN94 | Female | 24 years | Congenital Thrombotic thrombocytopenic purpura | Negative |
| WHN95 | Male | 57 years | Leukoencephalopathy | Negative |
| WHN96 | Male | 3 years | Glomerulosclerosis disease | Negative |
| WHN97 | Female | 11 years | Glomerulosclerosis disease | Negative |
| WHN98 | Female | 5 years | Nephropathy | Negative |
| WHN99 | Male | 4 years | Immune system diseases | Negative |
| WHN100 | Female | 41 years | Spinocerebellar Ataxia | Negative |
| WHN101 | Male | 42 years | Hereditary muscular disease | Negative |
| WHN102 | Male | 13 years | Glomerulosclerosis disease | Negative |
| WHN103 | Female | 25 years | Glomerulosclerosis disease | Negative |
| WHN104 | Male | 13 years | Glomerulosclerosis disease | Negative |
| WHN105 | - | - | Nephropathy | Negative |
| WHN106 | Female | 36 years | Cerebrovascular disease | Negative |
| WHN107 | Male | 52 years | Renal Tubular disease | Negative |
| WHN108 | Female | 76 years | Nephropathy | Negative |
| WHN109 | Female | 49 years | Nephropathy | Negative |
| WHN110 | Male | 23 years | Nephropathy | Negative |
| WHN111 | Male | 17 years | Glomerulosclerosis disease | Negative |
| WHN112 | Male | 26 years | Albinism | Negative |
| WHN113 | Female | - | Albinism | Negative |
| WHN114 | Male | 9 years | Glomerulosclerosis disease | Negative |
| WHN115 | Male | 37 years | - | Negative |
| WHN116 | Female | 36 years | - | Negative |
| WHN117 | Male | 2 years | - | Negative |
| WHN118 | Female | 12 years | Glomerulosclerosis disease | Negative |
| WHN119 | Male | 40 years | - | Negative |
| WHN120 | Female | 39 years | - | Negative |
| WHN121 | Male | 10 years | Glomerulosclerosis disease | Negative |
| WHN122 | Female | 5 years | Glomerulosclerosis disease | Negative |
| WHN123 | Male | 44 years | Hereditary peripheral neuropathy | Negative |
| WHN124 | Male | 13 years | Renal Tubular disease | Negative |
| WHN125 | Male | 5 years | Glomerulosclerosis disease | Negative |
| WHN126 | Male | 1 months | Renal Tubular disease | Negative |
| WHN127 | Male | 9 years | Glomerulosclerosis disease | Negative |
| WHN128 | Male | 4 years | Renal Tubular disease | Negative |
| WHN129 | Male | 9 years | Glomerulosclerosis disease | Negative |
| WHN130 | Male | 8 months | Glomerulosclerosis disease | Negative |
| WHN131 | Male | 43 years | Hereditary peripheral neuropathy | Negative |
| WHN132 | Male | 3 years | Glomerulosclerosis disease | Negative |
| WHN133 | Male | 33 years | Hereditary muscular disease | Negative |
| WHN134 | Male | 28 years | Hereditary muscular disease | Negative |
| WHN135 | Female | 17 years | Hereditary muscular disease | Negative |
| WHN136 | Male | 9 years | Glomerulosclerosis disease | Negative |
| WHN137 | Male | 8 years | Glomerulosclerosis disease | Negative |
| WHN138 | Male | 51 years | Aneurysm disease | Negative |
| WHN139 | Female | 9 years | Renal Tubular disease | Negative |
| WHN140 | Female | 9 years | Glomerulosclerosis disease | Negative |
| WHN141 | Female | 13 years | Glomerulosclerosis disease | Negative |
| WHN142 | Male | 3 years | Glomerulosclerosis disease | Negative |
| WHN143 | Male | 45 years | Charcot-Marie-Tooth disease/ | Negative |
| WHN144 | Male | 7 years | Renal Tubular disease | Negative |
| WHN145 | Male | 61 years | Hereditary muscular disease | Negative |
| WHN146 | Male | 7 years | Nephropathy | Negative |
| WHN147 | Female | 12 years | Renal Tubular disease | Negative |
| WHN148 | Male | 20 years | Hereditary muscular disease/Charcot-Marie-Tooth disease | Negative |
| WHN149 | Male | 10 years | Renal Tubular disease | Negative |
| WHN150 | Male | 3 years | Hereditary ataxia | Negative |
| WHN151 | Female | 28 years | Hereditary muscular disease | Negative |

**3. Gene List**

A2M, AAAS, AADAT, AANAT, AARS, AARS2, AASS, ABAT, ABCA1, ABCA12, ABCA3, ABCA4, ABCA9, ABCB1, ABCB10, ABCB11, ABCB4, ABCB5, ABCB6, ABCB7, ABCB8, ABCC1, ABCC12, ABCC2, ABCC6, ABCC8, ABCC9, ABCD1, ABCD2, ABCD3, ABCD4, ABCF2, ABCG5, ABCG8, ABHD10, ABHD11, ABHD12, ABHD5, ABL1, ABO, ACAA1, ACAA2, ACACA, ACACB, ACAD10, ACAD11, ACAD8, ACAD9, ACADL, ACADM, ACADS, ACADSB, ACADVL, ACAN, ACAT1, ACAT2, ACBD5, ACE, ACE2, ACHE, ACLY, ACN9, ACO1, ACO2, ACOT13, ACOT2, ACOT7, ACOT9, ACOX1, ACOX3, ACP5, ACP6, ACRC, ACSF2, ACSF3, ACSL1, ACSL4, ACSL5, ACSL6, ACSM1, ACSM2A, ACSM3, ACSM4, ACSM5, ACSS1, ACTA1, ACTA2, ACTB, ACTC1, ACTG1, ACTG2, ACTN1, ACTN2, ACTN4, ACTRT1, ACVR1, ACVR2B, ACVRL1, ACY1, ACYP2, ADA, ADAM17, ADAM9, ADAMTS10, ADAMTS13, ADAMTS17, ADAMTS2, ADAMTSL2, ADAMTSL4, ADAR, ADAT3, ADCK1, ADCK2, ADCK3, ADCK4, ADCK5, ADH1C, ADHFE1, ADK, ADNP, ADO, ADRA2A, ADRB1, ADRB2, ADRB3, ADSL, AFF2, AFG3L2, AFP, AGA, AGK, AGL, AGMAT, AGPAT2, AGPAT5, AGPS, AGR2, AGRN, AGT, AGTR1, AGTR2, AGXT, AGXT2, AGXT2L2, AHCY, AHDC1, AHI1, AICDA, AIFM1, AIFM2, AIFM3, AIM1, AIMP1, AIP, AIPL1, AIRE, AK1, AK2, AK3, AKAP1, AKAP10, AKAP14, AKAP17A, AKAP4, AKAP9, AKR1B15, AKR1D1, AKR7A2, AKT1, AKT2, AKT3, ALAD, ALAS1, ALAS2, ALB, ALDH18A1, ALDH1B1, ALDH1L1, ALDH1L2, ALDH2, ALDH3A2, ALDH4A1, ALDH5A1, ALDH6A1, ALDH7A1, ALDH9A1, ALDOA, ALDOB, ALG1, ALG10, ALG11, ALG12, ALG13, ALG14, ALG2, ALG3, ALG6, ALG8, ALG9, ALK, ALKBH7, ALMS1, ALOX12B, ALOXE3, ALPL, ALS2, ALX1, ALX3, ALX4, AMACR, AMELX, AMER1, AMH, AMHR2, AMMECR1, AMN, AMOT, AMPD1, AMPD3, AMT, ANG, ANGPTL3, ANGPTL4, ANK1, ANK2, ANK3, ANKH, ANKRD1, ANKRD11, ANKRD26, ANKS6, ANLN, ANO10, ANO5, ANO6, ANTXR2, AP1S1, AP1S2, AP2S1, AP3B1, AP4B1, AP4E1, AP4M1, AP4S1, AP5Z1, APC, APCDD1, APEX2, APLN, APOA1, APOA1BP, APOA2, APOA5, APOB, APOC2, APOC3, APOE, APOL1, APOO, APOOL, APOPT1, APP, APRT, APTX, AQP2, AQP7, AR, ARAF, ARFGEF2, ARG1, ARG2, ARHGAP24, ARHGAP31, ARHGAP36, ARHGAP4, ARHGAP6, ARHGDIA, ARHGEF10, ARHGEF15, ARHGEF2, ARHGEF6, ARHGEF9, ARID1A, ARID1B, ARL13A, ARL13B, ARL2BP, ARL6, ARMC1, ARMC10, ARMC4, ARMCX1, ARMCX2, ARMCX3, ARMCX4, ARMCX5, ARMCX5-GPRASP2, ARMCX6, ARMS2, ARR3, ARSA, ARSB, ARSD, ARSE, ARSF, ARSH, ARX, AS3MT, ASAH1, ASAH2, ASB11, ASB12, ASB9, ASCL1, ASF1B, ASL, ASPA, ASPM, ASS1, ASXL1, ATAD1, ATAD3A, ATAD3B, ATCAY, ATG16L1, ATG4A, ATIC, ATL1, ATM, ATN1, ATP10D, ATP11C, ATP13A2, ATP1A2, ATP1A3, ATP1B4, ATP2A1, ATP2A2, ATP2B2, ATP2B3, ATP2C1, ATP5A1, ATP5B, ATP5C1, ATP5D, ATP5E, ATP5F1, ATP5G1, ATP5G2, ATP5G3, ATP5H, ATP5I, ATP5J, ATP5J2, ATP5L, ATP5O, ATP5S, ATP5SL, ATP6AP1, ATP6AP2, ATP6V0A2, ATP6V0A4, ATP6V1B1, ATP7A, ATP7B, ATP8A2, ATP8B1, ATPAF1, ATPAF2, ATPIF1, ATR, ATRIP, ATRX, ATXN1, ATXN10, ATXN2, ATXN3, ATXN3L, ATXN7, ATXN8OS, AUH, AURKAIP1, AURKC, AUTS2, AVP, AVPR1A, AVPR2, AWAT1, AWAT2, AXIN2, AZF1, AZGP1, B3GALNT2, B3GALT6, B3GALTL, B3GAT3, B3GNT1, B4GALNT1, B4GALT1, B4GALT7, B9D1, B9D2, BAAT, BAD, BAG3, BAK1, BANF1, BANK1, BAP1, BARD1, BAX, BBIP1, BBOX1, BBS1, BBS10, BBS12, BBS2, BBS4, BBS5, BBS7, BBS9, BCAP31, BCAT1, BCAT2, BCHE, BCKDHA, BCKDHB, BCKDK, BCL2, BCL2L13, BCOR, BCORL1, BCS1L, BCYRN1, BDH1, BDKRB1, BDNF, BEAN1, BEND2, BEST1, BEX1, BEX2, BEX4, BEX5, BFSP1, BFSP2, BGN, BHLHB9, BHMT, BICC1, BICD2, BID, BIN1, BIRC5, BLK, BLM, BLNK, BLOC1S1, BLOC1S3, BLOC1S6, BMP1, BMP15, BMP2, BMP4, BMPER, BMPR1A, BMPR1B, BMPR2, BMX, BOLA1, BOLA3, BPHL, BRAF, BRAT1, BRCA1, BRCA2, BRCC3, BRD2, BRIP1, BRS3, BRWD3, BSCL2, BSND, BTD, BTK, BUB1B, C10orf10, C10orf11, C10orf2, C12orf57, C12orf65, C14orf159, C14orf2, C15orf41, C15orf48, C17orf89, C19orf12, C19orf70, C1GALT1C1, C1orf106, C1QA, C1QB, C1QBP, C1QC, C1QTNF5, C1R, C1S, C2, C20orf24, C21orf2, C21orf33, C21orf59, C2orf47, C2orf71, C3, C3orf58, C4A, C4B, C4orf26, C5, C5orf42, C6, C6orf136, C6orf203, C6orf57, C7, C7orf55, C8A, C8B, C8orf37, C9, C9orf72, CA12, CA2, CA4, CA5A, CA5B, CA5BP1, CA8, CABP2, CABP4, CACNA1A, CACNA1B, CACNA1C, CACNA1D, CACNA1F, CACNA1G, CACNA1H, CACNA1S, CACNA2D1, CACNA2D4, CACNB2, CACNB4, CACNG2, CALM1, CALM2, CALR, CALR3, CAMTA1, CANT1, CAPN10, CAPN3, CAPN5, CAPN6, CARD11, CARD14, CARD9, CARKD, CARS2, CARTPT, CASC5, CASK, CASP10, CASP8, CASQ2, CASR, CAT, CATSPER1, CATSPER2, CAV1, CAV3, CBL, CBR4, CBS, CC2D1A, CC2D2A, CCBE1, CCBL2, CCDC103, CCDC11, CCDC114, CCDC120, CCDC136, CCDC151, CCDC160, CCDC164, CCDC22, CCDC28B, CCDC39, CCDC40, CCDC50, CCDC51, CCDC58, CCDC65, CCDC73, CCDC78, CCDC8, CCDC88C, CCDC90A, CCDC90B, CCL2, CCL21, CCM2, CCNB3, CCNO, CCR5, CCR6, CCT5, CCT7, CD151, CD19, CD2, CD247, CD27, CD28, CD2AP, CD320, CD36, CD3D, CD3E, CD3G, CD40, CD40LG, CD46, CD58, CD59, CD79A, CD79B, CD81, CD8A, CD96, CD99L2, CDAN1, CDC20, CDC20B, CDC42BPA, CDC42BPB, CDC42BPG, CDC6, CDC73, CDCA3, CDCA8, CDH1, CDH13, CDH15, CDH23, CDH3, CDHR1, CDK16, CDK4, CDK5RAP2, CDK6, CDKL5, CDKN1A, CDKN1B, CDKN1C, CDKN2A, CDKN2B, CDKN2C, CDKN3, CDON, CDR1, CDSN, CDT1, CDX4, CEACAM16, CEBPA, CEBPE, CECR5, CEL, CELSR2, CENPE, CENPF, CENPI, CENPJ, CENPM, CENPVP1, CENPVP2, CEP135, CEP152, CEP164, CEP290, CEP41, CEP55, CEP57, CEP63, CERK, CERKL, CERS1, CERS3, CETN2, CETP, CFB, CFC1, CFD, CFH, CFHR1, CFHR3, CFHR4, CFHR5, CFI, CFL2, CFP, CFTR, CHAT, CHCHD1, CHCHD10, CHCHD2, CHCHD3, CHCHD4, CHCHD7, CHD1, CHD1L, CHD2, CHD7, CHD8, CHDC2, CHDH, CHEK1, CHEK2, CHGB, CHIC1, CHIC2, CHIT1, CHKB, CHL1, CHM, CHMP1A, CHMP2B, CHMP4B, CHN1, CHRDL1, CHRM3, CHRNA1, CHRNA2, CHRNA3, CHRNA4, CHRNA7, CHRNB1, CHRNB2, CHRND, CHRNE, CHRNG, CHST14, CHST3, CHST6, CHST7, CHST8, CHSY1, CIB2, CIDEC, CIITA, CIRH1A, CISD1, CISD2, CITED1, CITED2, CIZ1, CKB, CKM, CKMT1A, CKMT1B, CKMT2, CLCF1, CLCN1, CLCN2, CLCN4, CLCN5, CLCN7, CLCNKA, CLCNKB, CLDN1, CLDN14, CLDN16, CLDN19, CLDN2, CLIC2, CLIC4, CLN3, CLN5, CLN6, CLN8, CLPB, CLPP, CLPX, CLRN1, CLU, CLYBL, CMC1, CMC2, CMC4, CNBP, CNGA1, CNGA2, CNGA3, CNGB1, CNGB3, CNKSR2, CNNM2, CNNM4, CNR1, CNR2, CNTN1, CNTN2, CNTN3, CNTN4, CNTNAP2, CNTNAP5, COA5, COA6, COCH, COG1, COG4, COG5, COG6, COG7, COG8, COL10A1, COL11A1, COL11A2, COL12A1, COL17A1, COL18A1, COL1A1, COL1A2, COL2A1, COL3A1, COL4A1, COL4A2, COL4A3, COL4A4, COL4A5, COL4A6, COL5A1, COL5A2, COL6A1, COL6A2, COL6A3, COL7A1, COL8A2, COL9A1, COL9A2, COL9A3, COLEC11, COLQ, COMP, COMT, COMTD1, COQ10A, COQ10B, COQ2, COQ3, COQ4, COQ5, COQ6, COQ7, COQ9, CORIN, CORO1A, COX10, COX11, COX14, COX15, COX16, COX17, COX18, COX19, COX20, COX4I1, COX4I2, COX5A, COX5B, COX6A1, COX6A2, COX6B1, COX6B2, COX6C, COX7A1, COX7A2, COX7A2L, COX7B, COX7B2, COX7C, COX8A, COX8C, CP, CPA1, CPA6, CPOX, CPS1, CPT1A, CPT1B, CPT1C, CPT2, CPXCR1, CR1, CR2, CRADD, CRAT, CRB1, CRBN, CREB3L1, CREBBP, CRELD1, CRH, CRLF1, CRLF2, CRLS1, CROT, CRTAP, CRX, CRY1, CRYAA, CRYAB, CRYBA1, CRYBA2, CRYBA4, CRYBB1, CRYBB2, CRYBB3, CRYGA, CRYGB, CRYGC, CRYGD, CRYGS, CRYL1, CRYM, CS, CSAG1, CSAG3, CSF1R, CSF2RA, CSF2RB, CSF3, CSF3R, CSNK1D, CSRP3, CST3, CSTA, CSTB, CSTF2, CT45A1, CT45A2, CT45A3, CT45A4, CT45A5, CT45A6, CT55, CT83, CTAG1A, CTAG1B, CTAG2, CTC1, CTCF, CTDP1, CTF1, CTH, CTNNA3, CTNNB1, CTNS, CTPS1, CTPS2, CTRC, CTSA, CTSC, CTSD, CTSF, CTSK, CTU1, CUBN, CUL3, CUL4B, CUL7, CWF19L1, CX3CR1, CXCL12, CXCR3, CXCR4, CXorf21, CXorf22, CXorf23, CXorf28, CXorf30, CXorf31, CXorf36, CXorf38, CXorf40A, CXorf40B, CXorf49, CXorf49B, CXorf51A, CXorf51B, CXorf56, CXorf57, CXorf58, CXorf65, CXorf66, CXorf67, CXXC1P1, CYB5A, CYB5B, CYB5R1, CYB5R2, CYB5R3, CYBA, CYBB, CYC1, CYCS, CYLC1, CYLD, CYP11A1, CYP11B1, CYP11B2, CYP17A1, CYP19A1, CYP1A1, CYP1A2, CYP1B1, CYP21A2, CYP24A1, CYP26C1, CYP27A1, CYP27B1, CYP2A13, CYP2A6, CYP2A7, CYP2B6, CYP2C18, CYP2C19, CYP2C8, CYP2C9, CYP2D6, CYP2E1, CYP2F1, CYP2J2, CYP2R1, CYP2S1, CYP2U1, CYP2W1, CYP3A4, CYP3A43, CYP3A5, CYP3A7, CYP4F2, CYP4F22, CYP4V2, CYP7A1, CYP7B1, CYSLTR1, D2HGDH, DACH2, DACT2, DAG1, DAGLA, DAP3, DARS2, DAZ1, DBH, DBT, DCAF12L1, DCAF12L2, DCAF17, DCAF5, DCAF8L1, DCAF8L2, DCAKD, DCHS1, DCLK2, DCLRE1C, DCN, DCTN1, DCX, DDAH1, DDB2, DDC, DDHD1, DDHD2, DDOST, DDR2, DDX11, DDX25, DDX26B, DDX28, DDX3X, DDX53, DECR1, DEPDC5, DES, DFNA5, DFNB31, DFNB59, DGAT2L6, DGKE, DGKK, DGUOK, DHCR24, DHCR7, DHDDS, DHFR, DHH, DHODH, DHRS1, DHRS4, DHTKD1, DHX29, DIABLO, DIAPH1, DIAPH2, DIAPH3, DICER1, DIP2B, DIRAS3, DIS3L2, DISC1, DISP1, DKC1, DLAT, DLD, DLG3, DLGAP2, DLGAP5, DLL1, DLL3, DLST, DLX3, DLX5, DMD, DMGDH, DMP1, DMPK, DMRTC1, DMRTC1B, DNA2, DNAAF1, DNAAF2, DNAAF3, DNAH11, DNAH5, DNAI1, DNAI2, DNAJA3, DNAJB2, DNAJB6, DNAJC11, DNAJC15, DNAJC19, DNAJC27, DNAJC30, DNAJC4, DNAJC5, DNAJC6, DNAL1, DNASE1, DNASE1L1, DNM1, DNM1L, DNM2, DNMT1, DNMT3A, DNMT3B, DOCK11, DOCK4, DOCK6, DOCK8, DOK7, DOLK, DPAGT1, DPM1, DPM2, DPM3, DPP10, DPP6, DPY19L2, DPYD, DPYS, DRD2, DRD3, DRD5, DRG2, DRP2, DSC1, DSC2, DSC3, DSE, DSG1, DSG2, DSG3, DSG4, DSP, DSPP, DST, DTL, DTNA, DTNBP1, DUOX2, DUOXA2, DUS2, DUSP16, DUSP21, DUSP26, DUSP6, DUSP9, DUT, DUX4, DYM, DYNC1H1, DYNC2H1, DYNLT3, DYRK1A, DYSF, DYX1C1, E2F1, E2F3, EARS2, EBP, ECE1, ECEL1, ECH1, ECHDC3, ECHS1, ECI1, ECI2, ECSIT, EDA, EDA2R, EDAR, EDARADD, EDN1, EDN3, EDNRB, EEF1A2, EEF2, EEFSEC, EFEMP1, EFEMP2, EFHC1, EFHC2, EFHD1, EFNB1, EFTUD2, EGF, EGFL6, EGFR, EGLN1, EGR1, EGR2, EHHADH, EHMT1, EIF1AX, EIF2AK3, EIF2AK4, EIF2B1, EIF2B2, EIF2B3, EIF2B4, EIF2B5, EIF2S3, EIF4G1, ELAC2, ELANE, ELF4, ELK1, ELMOD2, ELMOD3, ELN, ELOVL4, ELOVL5, ELP4, EMC1, EMC2, EMD, EMP2, EMX2, EN2, ENAM, ENDOG, ENG, ENO1, ENO3, ENOX2, ENPP1, EOGT, EOMES, EP300, EPAS1, EPB41, EPB41L1, EPB42, EPCAM, EPG5, EPHA2, EPM2A, EPOR, ERAP2, ERAS, ERBB2, ERBB3, ERCC1, ERCC2, ERCC3, ERCC4, ERCC5, ERCC6, ERCC6L, ERCC8, ERF, ERLIN2, ERMARD, ESCO2, ESPN, ESR1, ESR2, ESRRB, ESX1, ETFA, ETFB, ETFDH, ETHE1, ETV4, ETV5, EVC, EVC2, EXO1, EXOG, EXOSC3, EXPH5, EXT1, EXT2, EYA1, EYA4, EYS, EZH2, F10, F11, F12, F13A1, F13B, F2, F5, F7, F8, F8A1, F8A2, F8A3, F9, FA2H, FAAH, FAAH2, FADD, FADS1, FAH, FAHD1, FAHD2A, FAM104B, FAM111A, FAM120C, FAM122B, FAM122C, FAM126A, FAM127A, FAM127B, FAM127C, FAM133A, FAM134B, FAM136A, FAM155B, FAM156A, FAM156B, FAM161A, FAM161B, FAM162A, FAM175A, FAM189A2, FAM199X, FAM20A, FAM20C, FAM210A, FAM213A, FAM223A, FAM223B, FAM226A, FAM226B, FAM3A, FAM45B, FAM46D, FAM47A, FAM47B, FAM47C, FAM50A, FAM58A, FAM65C, FAM83H, FAM9A, FAM9B, FAM9C, FAN1, FANCA, FANCB, FANCC, FANCD2, FANCE, FANCF, FANCG, FANCI, FANCL, FANCM, FAP, FARS2, FAS, FASLG, FASN, FASTK, FASTKD2, FAT4, FATE1, FBLN1, FBLN5, FBN1, FBN2, FBP1, FBXL4, FBXO31, FBXO38, FBXO7, FBXW11, FBXW4, FBXW7, FCGR3A, FCN3, FDPS, FDX1, FDX1L, FDXR, FECH, FERMT1, FERMT3, FGA, FGB, FGD1, FGD4, FGF10, FGF13, FGF13-AS1, FGF14, FGF16, FGF17, FGF20, FGF23, FGF3, FGF8, FGF9, FGFR1, FGFR2, FGFR3, FGFR4, FGG, FH, FHL1, FHL2, FIG4, FIGF, FIGLA, FIP1L1, FIS1, FITM2, FKBP10, FKBP14, FKBP8, FKRP, FKTN, FLCN, FLG, FLNA, FLNB, FLNC, FLRT3, FLT3, FLT4, FLVCR1, FLVCR2, FMN1, FMN2, FMO3, FMR1, FMR1-AS1, FMR1NB, FOLH1, FOLR1, FOLR2, FOLR3, FOXA2, FOXC1, FOXC2, FOXE1, FOXE3, FOXF1, FOXG1, FOXH1, FOXI1, FOXL2, FOXM1, FOXN1, FOXO4, FOXP1, FOXP2, FOXP3, FOXR2, FOXRED1, FPGS, FPR1, FRAS1, FREM1, FREM2, FRMD3, FRMD7, FRMD8P1, FRMPD3, FRMPD4, FSCN2, FSHB, FSHR, FTCD, FTH1, FTHL17, FTL, FTMT, FTO, FTSJ1, FTSJ2, FTX, FUCA1, FUNDC1, FUNDC2, FUS, FUT2, FUZ, FXN, FXYD2, FYCO1, FZD4, G6PC, G6PC3, G6PD, GAA, GAB3, GABBR1, GABBR2, GABRA1, GABRA2, GABRA3, GABRA4, GABRA5, GABRA6, GABRB1, GABRB2, GABRB3, GABRD, GABRE, GABRG1, GABRG2, GABRG3, GABRP, GABRQ, GABRR1, GABRR2, GABRR3, GAD1, GAD2, GADD45GIP1, GADL1, GALC, GALE, GALK1, GALNS, GALNT12, GALNT2, GALNT3, GALT, GAMT, GAN, GARS, GAS1, GATA1, GATA2, GATA3, GATA4, GATA5, GATA6, GATAD1, GATAD2B, GATC, GATM, GBA, GBA2, GBAS, GBE1, GCAT, GCDH, GCH1, GCK, GCKR, GCLC, GCM2, GCNT2, GCSH, GDAP1, GDAP1L1, GDF1, GDF2, GDF3, GDF5, GDF6, GDF9, GDI1, GDNF, GDPD2, GEMIN8, GFAP, GFER, GFI1, GFI1B, GFM1, GFM2, GFPT1, GGCX, GH1, GHITM, GHR, GHRH, GHRHR, GHSR, GIF, GIGYF2, GIMAP5, GIPC3, GJA1, GJA3, GJA5, GJA8, GJB1, GJB2, GJB3, GJB4, GJB6, GJC1, GJC2, GJD2, GK, GK2, GLA, GLB1, GLDC, GLE1, GLI2, GLI3, GLIS2, GLIS3, GLMN, GLO1, GLOD4, GLOD5, GLRA1, GLRA2, GLRA4, GLRB, GLRX, GLRX2, GLRX5, GLS, GLS2, GLUD1, GLUD2, GLUL, GLYAT, GLYCTK, GM2A, GMPPA, GMPPB, GNA11, GNA14, GNAI2, GNAI3, GNAL, GNAO1, GNAQ, GNAS, GNAT1, GNAT2, GNB3, GNB4, GNE, GNG5, GNL3L, GNMT, GNPAT, GNPTAB, GNPTG, GNRH1, GNRHR, GNS, GORAB, GOSR2, GOT2, GP1BA, GP1BB, GP6, GP9, GPAM, GPC3, GPC4, GPC6, GPD1, GPD1L, GPD2, GPHN, GPI, GPIHBP1, GPKOW, GPM6B, GPR101, GPR112, GPR119, GPR125, GPR143, GPR173, GPR174, GPR179, GPR34, GPR50, GPR55, GPR56, GPR64, GPR82, GPR98, GPRASP1, GPRASP2, GPSM2, GPT2, GPX1, GPX4, GRB10, GREM1, GRHL2, GRHL3, GRHPR, GRIA3, GRIK2, GRIN1, GRIN2A, GRIN2B, GRIP1, GRIPAP1, GRK1, GRK5, GRM1, GRM6, GRN, GRPEL1, GRPEL2, GRPR, GRSF1, GRXCR1, GSN, GSPT2, GSR, GSS, GSTA1, GSTK1, GSTM1, GSTP1, GTF2H5, GTPBP10, GTPBP3, GTPBP8, GUCA1A, GUCA1B, GUCY1A3, GUCY2C, GUCY2D, GUCY2F, GUF1, GUSB, GYG1, GYG2, GYS1, GYS2, H19, H2AFB1, H2AFB2, H2AFB3, H2BFM, H2BFWT, H2BFXP, H6PD, HABP2, HADH, HADHA, HADHB, HAGH, HAL, HAMP, HAO2, HARS, HARS2, HAUS7, HAX1, HBA1, HBA2, HBB, HBD, HBE1, HBG1, HBG2, HBZ, HCCS, HCFC1, HCN1, HCN2, HCN3, HCN4, HDAC4, HDAC6, HDAC8, HDDC2, HDHD1, HDX, HEATR2, HEMK1, HEPACAM, HEPH, HERC2, HES7, HESX1, HEXA, HEXB, HFE, HFE2, HFM1, HGD, HGF, HGSNAT, HIBADH, HIBCH, HIGD1A, HIGD2A, HINT1, HINT2, HK1, HK2, HK3, HLA-B, HLA-DQA1, HLA-DQB1, HLA-DRB1, HLA-H, HLCS, HMBS, HMCN1, HMGB3, HMGCL, HMGCR, HMGCS2, HMGN5, HMX2, HNF1A, HNF1B, HNF4A, HNMT, HNRNPA1, HNRNPA2B1, HNRNPH2, HNRNPU, HOGA1, HOXA1, HOXA11, HOXA13, HOXA2, HOXB1, HOXB13, HOXD10, HOXD13, HOXD3, HP, HPD, HPDL, HPGD, HPRT1, HPS1, HPS3, HPS4, HPS5, HPS6, HPSE2, HR, HRAS, HRSP12, HS6ST1, HS6ST2, HS6ST2-AS1, HSCB, HSD11B1, HSD11B2, HSD17B10, HSD17B3, HSD17B4, HSD17B8, HSD3B1, HSD3B2, HSD3B7, HSDL1, HSDL2, HSF4, HSFX1, HSFX2, HSPA9, HSPB1, HSPB3, HSPB7, HSPB8, HSPD1, HSPE1, HSPG2, HTATSF1, HTR1A, HTR1B, HTR2A, HTR2B, HTR2C, HTR3A, HTR3B, HTR3C, HTR3E, HTR5A, HTR6, HTR7, HTRA1, HTRA2, HTT, HUWE1, HYAL1, HYDIN, HYLS1, HYMAI, HYPM, IARS2, IBA57, ICK, ICOS, ICT1, IDE, IDH1, IDH2, IDH3A, IDH3B, IDH3G, IDI1, IDS, IDUA, IER3IP1, IFITM5, IFNG, IFNGR1, IFNGR2, IFRD1, IFT122, IFT140, IFT172, IFT43, IFT80, IGBP1, IGF1, IGF1R, IGF2, IGFALS, IGFBP7, IGHMBP2, IGLL1, IGSF1, IHH, IKBKAP, IKBKB, IKBKG, IKZF1, IL10, IL10RA, IL10RB, IL11RA, IL12B, IL12RB1, IL13RA1, IL13RA2, IL17F, IL17RA, IL17RD, IL18RAP, IL1RAPL1, IL1RAPL2, IL1RN, IL2, IL21, IL23R, IL28B, IL2RA, IL2RB, IL2RG, IL36RN, IL6, IL6ST, IL7R, ILDR1, ILK, IMMP2L, IMMT, IMPAD1, IMPDH1, IMPG1, IMPG2, INE1, INE2, INF2, INGX, INPP5E, INPPL1, INS, INSIG2, INSL3, INSR, INVS, IQCB1, IQCE, IQSEC2, IRAK1, IRAK4, IREB2, IRF5, IRF6, IRF8, IRGM, IRS4, IRX4, IRX5, ISCA1, ISCA2, ISCU, ISG15, ISOC2, ISPD, ITCH, ITGA2, ITGA2B, ITGA3, ITGA6, ITGA7, ITGA8, ITGAM, ITGB1BP2, ITGB2, ITGB3, ITGB4, ITIH6, ITK, ITM2A, ITM2B, ITPA, ITPR1, IVD, IYD, JADE3, JAG1, JAK2, JAK3, JAM3, JPH2, JPH3, JPX, JRK, JUP, KAL1, KANK1, KANK2, KANSL1, KARS, KAT6B, KATNAL2, KBTBD13, KCNA1, KCNA4, KCNA5, KCNAB1, KCNC3, KCND1, KCND3, KCNE1, KCNE1L, KCNE2, KCNE3, KCNE4, KCNH2, KCNH5, KCNJ1, KCNJ10, KCNJ11, KCNJ12, KCNJ13, KCNJ2, KCNJ3, KCNJ5, KCNJ8, KCNK18, KCNK3, KCNK9, KCNMA1, KCNQ1, KCNQ1OT1, KCNQ2, KCNQ3, KCNQ4, KCNT1, KCNV2, KCTD10, KCTD13, KCTD7, KDM5C, KDM6A, KHK, KIAA0101, KIAA0141, KIAA0196, KIAA0226, KIAA1033, KIAA1210, KIAA1279, KIAA1549, KIAA1841, KIAA2022, KIF11, KIF1A, KIF1B, KIF1C, KIF20B, KIF21A, KIF22, KIF23, KIF2A, KIF4A, KIF5A, KIF5C, KIF7, KIRREL3, KISS1, KISS1R, KIT, KL, KLF1, KLF11, KLF8, KLHL10, KLHL13, KLHL15, KLHL3, KLHL34, KLHL4, KLHL40, KLHL41, KLHL7, KLHL9, KLK4, KLLN, KMT2A, KMT2D, KPTN, KRAS, KRBOX4, KRIT1, KRT1, KRT10, KRT12, KRT13, KRT14, KRT16, KRT17, KRT18, KRT2, KRT3, KRT4, KRT5, KRT6A, KRT6B, KRT6C, KRT71, KRT74, KRT8, KRT81, KRT83, KRT85, KRT86, KRT9, KYNU, L1CAM, L2HGDH, LACE1, LACTB, LACTB2, LAGE3, LAMA1, LAMA2, LAMA3, LAMA4, LAMB1, LAMB2, LAMB3, LAMC2, LAMC3, LAMP2, LAMTOR2, LAMTOR5, LANCL3, LAP3, LARGE, LARP7, LARS, LARS2, LAS1L, LBR, LCA5, LCAT, LCK, LCT, LDB3, LDHA, LDHAL6B, LDHB, LDHD, LDLR, LDLRAD1, LDLRAD4, LDLRAP1, LDOC1, LEFTY2, LEMD3, LEP, LEPR, LEPRE1, LEPREL1, LETM1, LETMD1, LFNG, LGALS2, LGI1, LHCGR, LHFPL1, LHFPL5, LHX1, LHX3, LHX4, LIAS, LIFR, LIG4, LIM2, LIMS2, LINC00086, LINC00087, LINC00629, LINC00630, LINC00633, LINC00684, LINS, LIPA, LIPC, LIPG, LIPH, LIPI, LIPN, LIPT1, LIPT2, LITAF, LMAN1, LMBR1, LMBRD1, LMF1, LMNA, LMNB1, LMNB2, LMO1, LMX1B, LONP1, LONRF3, LOR, LOXHD1, LOXL1, LPAR4, LPAR6, LPIN1, LPIN2, LPL, LRAT, LRBA, LRCH2, LRIT3, LRP2, LRP4, LRP5, LRP6, LRP8, LRPPRC, LRRC6, LRRC8A, LRRK2, LRSAM1, LRTOMT, LSM14A, LTA, LTBP2, LTBP3, LTBP4, LUZP4, LYRM1, LYRM2, LYRM4, LYRM5, LYRM7, LYST, LYZ, LZTFL1, LZTR1, MAB21L2, MAF, MAFB, MAGEL2, MAGI2, MAGIX, MAGT1, MAK, MALT1, MAMLD1, MAN1B1, MAN2B1, MANBA, MAOA, MAOB, MAP2K1, MAP2K2, MAP2K4P1, MAP3K1, MAP3K15, MAP7D2, MAP7D3, MAPK1, MAPK10, MAPT, 1-Mar, 2-Mar, MARK1, MARS, MARS2, MARVELD2, MASP1, MASP2, MASTL, MAT1A, MAT2B, MATN3, MATR3, MAVS, MAX, MBD1, MBD5, MBL2, MBNL3, MBP, MBTPS2, MC1R, MC2R, MC3R, MC4R, MCAT, MCCC1, MCCC2, MCEE, MCF2, MCFD2, MCIDAS, MCM10, MCM4, MCM6, MCOLN1, MCPH1, MCTS1, MCU, MCUR1, MDH1, MDH2, MDM2, ME1, ME2, ME3, MECP2, MECR, MED12, MED13L, MED14, MED17, MED23, MED25, MED28, MEF2A, MEF2C, MEFV, MEG3, MEGF10, MEGF8, MEN1, MEOX1, MERTK, MESP2, MEST, MET, METAP1D, METTL17, METTL23, MFN1, MFN2, MFRP, MFSD8, MGAT2, MGLL, MGME1, MGMT, MGP, MGST3, MIA3, MIAT, MIB1, MICU1, MICU2, MID1, MID1IP1, MID2, MIEF1, MINOS1, MIP, MIPEP, MIR105-1, MIR105-2, MIR106A, MIR1184-1, MIR1184-2, MIR1184-3, MIR1256, MIR1264, MIR1277, MIR1298, MIR1468, MIR17HG, MIR182, MIR183, MIR188, MIR18B, MIR1911, MIR1912, MIR19B2, MIR20B, MIR2114, MIR221, MIR222, MIR223, MIR23C, MIR3202-1, MIR3202-2, MIR320D2, MIR362, MIR363, MIR3690, MIR374A, MIR374B, MIR374C, MIR380, MIR384, MIR3978, MIR421, MIR424, MIR4328, MIR4329, MIR4330, MIR448, MIR450A1, MIR450A2, MIR450B, MIR452, MIR4536-1, MIR4767, MIR4768, MIR4769, MIR4770, MIR500A, MIR500B, MIR501, MIR502, MIR503, MIR504, MIR505, MIR506, MIR507, MIR508, MIR509-1, MIR509-2, MIR509-3, MIR510, MIR514A1, MIR514A2, MIR514A3, MIR514B, MIR532, MIR542, MIR545, MIR651, MIR660, MIR664B, MIR676, MIR718, MIR764, MIR766, MIR767, MIR888, MIR890, MIR891A, MIR891B, MIR892A, MIR892B, MIR92A2, MIR934, MIR96, MIR98, MIRLET7F2, MITF, MKKS, MKS1, MLC1, MLH1, MLH3, MLPH, MLXIPL, MLYCD, MMAA, MMAB, MMACHC, MMADHC, MMGT1, MMP1, MMP13, MMP2, MMP20, MMP9, MN1, MNX1, MOBP, MOCOS, MOCS1, MOCS2, MOCS3, MOGS, MORC4, MORF4L2, MORF4L2-AS1, MOSPD1, MOSPD2, MPC1, MPC1L, MPC2, MPDU1, MPDZ, MPI, MPL, MPLKIP, MPO, MPP1, MPST, MPV17, MPV17L, MPV17L2, MPZ, MR1, MRAP, MRAP2, MRAS, MRE11A, MRM1, MRPL1, MRPL10, MRPL11, MRPL12, MRPL13, MRPL14, MRPL15, MRPL16, MRPL17, MRPL18, MRPL19, MRPL2, MRPL20, MRPL21, MRPL22, MRPL23, MRPL24, MRPL27, MRPL28, MRPL3, MRPL30, MRPL32, MRPL33, MRPL34, MRPL35, MRPL36, MRPL37, MRPL38, MRPL39, MRPL4, MRPL40, MRPL41, MRPL42, MRPL43, MRPL44, MRPL45, MRPL46, MRPL47, MRPL48, MRPL49, MRPL50, MRPL51, MRPL52, MRPL53, MRPL54, MRPL55, MRPL57, MRPL9, MRPS10, MRPS11, MRPS12, MRPS14, MRPS15, MRPS16, MRPS17, MRPS18A, MRPS18B, MRPS18C, MRPS2, MRPS21, MRPS22, MRPS23, MRPS24, MRPS25, MRPS26, MRPS27, MRPS28, MRPS30, MRPS31, MRPS33, MRPS34, MRPS35, MRPS36, MRPS5, MRPS6, MRPS7, MRPS9, MRRF, MRS2, MS4A1, MSH2, MSH3, MSH6, MSL3, MSMO1, MSN, MSR1, MSRA, MSRB2, MSRB3, MSTN, MSX1, MSX2, MTAP, MTCH1, MTCH2, MTCP1, MTERF, MTERFD1, MTERFD3, MTFMT, MTFP1, MTFR1, MTG1, MTG2, MTHFD1, MTHFD1L, MTHFD2, MTHFD2L, MTHFR, MTHFS, MTIF2, MTIF3, MTM1, MTMR1, MTMR14, MTMR2, MTMR8, MTO1, MTOR, MTPAP, MTR, MTRF1, MTRF1L, MTRNR2L10, MTRR, MTTP, MTX1, MTX2, MUC1, MUC5B, MUL1, MUM1L1, MURC, MUSK, MUT, MUTYH, MVK, MXRA5, MYBPC1, MYBPC3, MYC, MYCN, MYD88, MYF6, MYH1, MYH10, MYH11, MYH14, MYH2, MYH3, MYH6, MYH7, MYH8, MYH9, MYL10, MYL2, MYL3, MYLK, MYLK2, MYO15A, MYO1A, MYO1C, MYO1E, MYO1F, MYO3A, MYO5A, MYO5B, MYO6, MYO7A, MYOC, MYOCD, MYOM1, MYOT, MYOZ2, MYPN, NAA10, NAGA, NAGLU, NAGPA, NAGS, NAIP, NANOS1, NAP1L2, NAP1L3, NAP1L6, NAPB, NARS, NARS2, NAT1, NAT2, NBAS, NBEAL2, NBN, NCAN, NCF1, NCF2, NCF4, NCOA4, NDE1, NDN, NDP, NDRG1, NDST1, NDUFA1, NDUFA10, NDUFA11, NDUFA12, NDUFA13, NDUFA2, NDUFA3, NDUFA4, NDUFA4L2, NDUFA5, NDUFA6, NDUFA7, NDUFA8, NDUFA9, NDUFAB1, NDUFAF1, NDUFAF2, NDUFAF3, NDUFAF4, NDUFAF5, NDUFAF6, NDUFAF7, NDUFB1, NDUFB10, NDUFB11, NDUFB2, NDUFB3, NDUFB4, NDUFB5, NDUFB6, NDUFB7, NDUFB8, NDUFB9, NDUFC1, NDUFC2, NDUFS1, NDUFS2, NDUFS3, NDUFS4, NDUFS5, NDUFS6, NDUFS7, NDUFS8, NDUFV1, NDUFV2, NDUFV3, NEB, NEBL, NEDD4L, NEFH, NEFL, NEGR1, NEK1, NEK2, NEK8, NEU1, NEU4, NEUROD1, NEUROG3, NEXN, NF1, NF2, NFIX, NFKB2, NFKBIA, NFS1, NFU1, NFXL1, NGF, NGFRAP1, NGLY1, NHEJ1, NHLRC1, NHP2, NHS, NHSL2, NID1, NIF3L1, NIN, NIPA1, NIPAL4, NIPBL, NIPSNAP1, NIPSNAP3A, NIPSNAP3B, NIT1, NKAP, NKAPP1, NKRF, NKX2-1, NKX2-3, NKX2-5, NKX2-6, NKX3-2, NLGN3, NLGN4X, NLN, NLRP1, NLRP12, NLRP14, NLRP2, NLRP3, NLRP7, NLRX1, NME1, NME2, NME3, NME4, NME6, NME8, NMNAT1, NNT, NOA1, NOBOX, NOD2, NODAL, NOG, NOL3, NONO, NOP10, NOP56, NOS1AP, NOS3, NOTCH1, NOTCH2, NOTCH3, NOX1, NPC1, NPC2, NPHP1, NPHP3, NPHP4, NPHS1, NPHS2, NPL, NPM1, NPPA, NPR2, NR0B1, NR1I2, NR1I3, NR2E1, NR2E3, NR2F1, NR3C2, NR5A1, NRAS, NRCAM, NRD1, NRF1, NRK, NRL, NRTN, NRXN1, NRXN2, NSD1, NSDHL, NSMF, NSUN2, NT5C, NT5C3A, NT5DC3, NT5M, NTF4, NTHL1, NTNG1, NTRK1, NTRK2, NUBPL, NUDT1, NUDT10, NUDT11, NUDT13, NUDT19, NUDT2, NUDT8, NUP155, NUP62, NUP62CL, NUSAP1, NXF2, NXF2B, NXF3, NXF4, NXF5, NXT2, NYX, OAT, OAZ1, OBSCN, OBSL1, OCA2, OCIAD1, OCLN, OCRL, OFD1, OGDH, OGDHL, OGG1, OGT, OMA1, OPA1, OPA3, OPHN1, OPN1LW, OPN1MW, OPN1MW2, OPN1SW, OPRM1, OPTN, OR13G1, OR13H1, ORAI1, ORC1, ORC4, ORC6, OSGEPL1, OSTM1, OTC, OTOA, OTOF, OTOG, OTOGL, OTUD4, OTUD5, OTUD6A, OTX2, OXA1L, OXCT1, OXCT2, OXLD1, OXNAD1, OXR1, OXSM, OXTR, P2RX2, P2RX7, P2RY10, P2RY12, P2RY4, PABPC1L2A, PABPC1L2B, PABPC5, PABPN1, PACRG, PACS1, PAFAH1B1, PAGE1, PAGE2, PAGE2B, PAGE3, PAGE4, PAGE5, PAH, PAK3, PAK7, PALB2, PALLD, PAM16, PANK2, PAPSS2, PARK2, PARK7, PARL, PARP1, PARS2, PASD1, PAX2, PAX3, PAX4, PAX5, PAX6, PAX8, PAX9, PBDC1, PBK, PBX1, PC, PCBD1, PCBD2, PCCA, PCCB, PCDH10, PCDH11X, PCDH15, PCDH19, PCDH9, PCK1, PCK2, PCM1, PCNT, PCSK1, PCSK1N, PCSK9, PCYT1A, PCYT1B, PDCD10, PDE10A, PDE11A, PDE12, PDE4D, PDE6A, PDE6B, PDE6C, PDE6D, PDE6G, PDE6H, PDE8B, PDGFRA, PDGFRB, PDHA1, PDHA2, PDHB, PDHX, PDK1, PDK2, PDK3, PDK4, PDLIM3, PDP1, PDP2, PDPR, PDSS1, PDSS2, PDX1, PDXK, PDYN, PDZD11, PDZD4, PDZD7, PEPD, PER2, PET112, PEX1, PEX10, PEX11A, PEX11B, PEX12, PEX13, PEX14, PEX16, PEX19, PEX2, PEX26, PEX3, PEX5, PEX5L, PEX6, PEX7, PFKFB1, PFKL, PFKM, PFN1, PGAM2, PGAM4, PGAP1, PGAP2, PGK1, PGM1, PGM3, PGRMC1, PGS1, PHACTR1, PHB, PHB2, PHC1, PHEX, PHEX-AS1, PHF6, PHF8, PHGDH, PHKA1, PHKA2, PHKA2-AS1, PHKB, PHKG2, PHOX2A, PHOX2B, PHYH, PHYHIPL, PHYKPL, PICALM, PIEZO1, PIEZO2, PIGA, PIGL, PIGM, PIGN, PIGO, PIGT, PIGV, PIGY, PIH1D3, PIK3C2A, PIK3CA, PIK3CD, PIK3R1, PIK3R2, PIK3R5, PIKFYVE, PIM2, PIN4, PINK1, PIP5K1B, PIP5K1C, PIR, PISD, PITPNM3, PITRM1, PITX1, PITX2, PITX3, PJA1, PKD1, PKD1L3, PKD2, PKHD1, PKLR, PKP1, PKP2, PKP4, PLA2G15, PLA2G5, PLA2G6, PLA2R1, PLAC1, PLAGL1, PLAU, PLCB1, PLCB4, PLCE1, PLCG2, PLEC, PLEKHG4, PLEKHG5, PLEKHM1, PLG, PLGRKT, PLIN1, PLK1, PLN, PLOD1, PLOD2, PLOD3, PLP1, PLP2, PLS3, PLTP, PLXNA3, PLXNB3, PMM2, PMP22, PMPCA, PMPCB, PMS1, PMS2, PNCK, PNKD, PNKP, PNLIP, PNMA3, PNMA5, PNMA6A, PNMT, PNN, PNP, PNPLA1, PNPLA2, PNPLA3, PNPLA4, PNPLA6, PNPO, PNPT1, POC1A, POC1B, POF1B, POLA1, POLD1, POLG, POLG2, POLH, POLR1C, POLR1D, POLR3A, POLR3B, POLRMT, POMC, POMGNT1, POMGNT2, POMK, POMP, POMT1, POMT2, PON3, POR, PORCN, POU1F1, POU3F4, POU4F3, PPA2, PPARG, PPARGC1A, PPARGC1B, PPEF1, PPIB, PPID, PPIF, PPM1B, PPM1D, PPM1K, PPOX, PPP1R2P9, PPP1R3B, PPP1R3F, PPP2R2B, PPT1, PPTC7, PQBP1, PRAF2, PRC1, PRCD, PRDM1, PRDM16, PRDM5, PRDM9, PRDX1, PRDX2, PRDX3, PRDX4, PRDX5, PRDX6, PRELID1, PRELID2, PREPL, PRF1, PRG4, PRICKLE1, PRICKLE2, PRICKLE3, PRICKLE4, PRKAA2, PRKAB1, PRKAB2, PRKAG2, PRKAR1A, PRKCD, PRKCG, PRKCQ, PRKCSH, PRKDC, PRKG1, PRKRA, PRKX, PRM1, PRM2, PRM3, PRNP, PROC, PRODH, PRODH2, PROK2, PROKR2, PROM1, PROP1, PROS1, PROSC, PRPF3, PRPF31, PRPF6, PRPF8, PRPH, PRPH2, PRPS1, PRPS2, PRR32, PRR4, PRR5L, PRRG1, PRRG3, PRRT2, PRRX1, PRSS1, PRSS12, PRSS2, PRSS35, PRSS56, PRX, PSAP, PSAT1, PSEN1, PSEN2, PSMA6, PSMB8, PSMC3IP, PSMD10, PSPH, PSTK, PSTPIP1, PTCD2, PTCD3, PTCH1, PTCHD1, PTEN, PTF1A, PTGES2, PTGR2, PTH, PTH1R, PTHLH, PTPLA, PTPMT1, PTPN11, PTPN22, PTPRC, PTPRO, PTPRQ, PTRF, PTRH1, PTRH2, PTS, PTTG1, PUS1, PVRL1, PXDN, PXMP2, PYCR1, PYGL, PYGM, PYY, QDPR, QRSL1, RAB11A, RAB11B, RAB11FIP5, RAB18, RAB1B, RAB20, RAB23, RAB24, RAB27A, RAB28, RAB32, RAB33A, RAB33B, RAB35, RAB39B, RAB3D, RAB3GAP1, RAB3GAP2, RAB40A, RAB40AL, RAB41, RAB4B, RAB7A, RAB8B, RAB9A, RAB9B, RAC2, RAD21, RAD50, RAD51, RAD51C, RAD51D, RAD54L, RAF1, RAG1, RAG2, RAI1, RAI2, RANBP2, RANGRF, RAP2C, RAPSN, RARA, RARB, RARS, RARS2, RASA1, RASGRP2, RAX, RAX2, RB1, RBBP7, RBBP8, RBFOX1, RBM10, RBM15, RBM20, RBM28, RBM3, RBM41, RBM8A, RBMX, RBMX2, RBMXL2, RBMXL3, RBP3, RBP4, RBPJ, RD3, RDH12, RDH13, RDH14, RDH5, RDX, RECQL4, REEP1, RELN, REN, RENBP, REPS2, RET, REXO2, RFK, RFT1, RFX5, RFX6, RFXANK, RFXAP, RGAG1, RGAG4, RGN, RGR, RGS9, RGS9BP, RHAG, RHBDF2, RHD, RHO, RHOA, RHOH, RHOT1, RHOT2, RHOXF1, RHOXF2, RHOXF2B, RIBC1, RILP, RIMS1, RIN2, RIPK4, RIPPLY1, RIT1, RLBP1, RLIM, RMDN1, RMDN2, RMDN3, RMND1, RMRP, RNASEH1, RNASEH2A, RNASEH2B, RNASEH2C, RNASEL, RNASET2, RNF113A, RNF128, RNF135, RNF168, RNF170, RNF213, RNF216, RNF8, RNMTL1, RNU4ATAC, RNU6-28P, ROBO2, ROBO3, ROGDI, ROM1, ROMO1, ROR2, RP1, RP1L1, RP2, RP9, RPA4, RPE65, RPGR, RPGRIP1, RPGRIP1L, RPIA, RPL10, RPL10A, RPL11, RPL19, RPL21, RPL26, RPL34, RPL35A, RPL36A, RPL36A-HNRNPH2, RPL37A, RPL39, RPL5, RPN2, RPS10, RPS14, RPS15A, RPS17, RPS19, RPS24, RPS26, RPS26P11, RPS4X, RPS6KA3, RPS6KA6, RPS7, RPS7P5, RPSA, RPUSD4, RRAGB, RRM2, RRM2B, RS1, RSAD1, RSPH1, RSPH4A, RSPH9, RSPO4, RTEL1, RTL1, RTN2, RTN4IP1, RTTN, RUNX1, RUNX2, RXFP2, RYR1, RYR2, S100G, SAA1, SACS, SAG, SAGE1, SALL1, SALL4, SAMD3, SAMD4A, SAMHD1, SAMM50, SAR1B, SARDH, SARS, SARS2, SASH3, SAT1, SATB2, SATL1, SBDS, SBF1, SBF2, SC5D, SCARB2, SCARNA23, SCARNA9L, SCCPDH, SCML1, SCML2, SCN10A, SCN11A, SCN1A, SCN1B, SCN2A, SCN2B, SCN3A, SCN3B, SCN4A, SCN4B, SCN5A, SCN7A, SCN8A, SCN9A, SCNN1A, SCNN1B, SCNN1G, SCO1, SCO2, SCP2, SDCCAG8, SDHA, SDHAF1, SDHAF2, SDHB, SDHC, SDHD, SDSL, SEC23A, SEC23B, SEC63, SECISBP2, SEMA3A, SEMA3E, SEMA4A, SEPN1, SEPSECS, 12-Sep, 4-Sep, 6-Sep, 9-Sep, SERAC1, SERHL2, SERPINA1, SERPINA10, SERPINA7, SERPINB6, SERPINC1, SERPIND1, SERPINE1, SERPINF1, SERPINF2, SERPING1, SERPINH1, SERPINI1, SERTAD1, SERTM1, SETBP1, SETX, SEZ6L, SF3B4, SFTPA1, SFTPA2, SFTPB, SFTPC, SFTPD, SFXN1, SFXN2, SFXN3, SFXN4, SFXN5, SGCA, SGCB, SGCD, SGCE, SGCG, SGSH, SH2B3, SH2D1A, SH3BGRL, SH3BP2, SH3KBP1, SH3PXD2B, SH3TC2, SHANK2, SHANK3, SHH, SHMT1, SHMT2, SHOC2, SHOX, SHOX2, SHROOM2, SHROOM4, SI, SIGMAR1, SIL1, SIM1, SIRT1, SIRT2, SIRT3, SIRT5, SIX1, SIX2, SIX3, SIX5, SIX6, SKI, SKIV2L, SLC10A3, SLC11A2, SLC12A1, SLC12A2, SLC12A3, SLC12A5, SLC12A6, SLC12A7, SLC16A1, SLC16A12, SLC16A2, SLC17A3, SLC17A5, SLC17A8, SLC19A1, SLC19A2, SLC19A3, SLC1A1, SLC1A3, SLC20A2, SLC22A12, SLC22A4, SLC22A5, SLC24A1, SLC24A4, SLC24A5, SLC25A1, SLC25A10, SLC25A11, SLC25A12, SLC25A13, SLC25A14, SLC25A15, SLC25A16, SLC25A17, SLC25A18, SLC25A19, SLC25A2, SLC25A20, SLC25A21, SLC25A22, SLC25A23, SLC25A24, SLC25A25, SLC25A26, SLC25A27, SLC25A28, SLC25A29, SLC25A3, SLC25A30, SLC25A31, SLC25A32, SLC25A33, SLC25A34, SLC25A35, SLC25A36, SLC25A37, SLC25A38, SLC25A39, SLC25A4, SLC25A40, SLC25A41, SLC25A42, SLC25A43, SLC25A44, SLC25A45, SLC25A46, SLC25A47, SLC25A48, SLC25A5, SLC25A51, SLC25A53, SLC25A5-AS1, SLC25A6, SLC26A2, SLC26A3, SLC26A4, SLC26A5, SLC26A8, SLC27A4, SLC27A5, SLC29A3, SLC2A1, SLC2A10, SLC2A2, SLC2A9, SLC30A10, SLC30A2, SLC30A6, SLC33A1, SLC34A1, SLC34A2, SLC34A3, SLC35A1, SLC35A2, SLC35A3, SLC35C1, SLC35D1, SLC35G2, SLC37A4, SLC38A5, SLC39A13, SLC39A4, SLC3A1, SLC40A1, SLC45A1, SLC45A2, SLC46A1, SLC4A1, SLC4A10, SLC4A11, SLC4A4, SLC4A5, SLC52A1, SLC52A2, SLC52A3, SLC5A1, SLC5A2, SLC5A5, SLC5A7, SLC6A14, SLC6A19, SLC6A20, SLC6A3, SLC6A4, SLC6A5, SLC6A8, SLC7A13, SLC7A3, SLC7A7, SLC7A9, SLC8A1, SLC9A1, SLC9A2, SLC9A3R1, SLC9A6, SLC9A7, SLC9A9, SLCO1B1, SLCO1B3, SLIRP, SLITRK1, SLITRK2, SLITRK4, SLMAP, SLMO1, SLMO2, SLURP1, SLX4, SMAD3, SMAD4, SMAD6, SMAD9, SMARCA1, SMARCA2, SMARCA4, SMARCAD1, SMARCAL1, SMARCB1, SMARCE1, SMC1A, SMC3, SMCHD1, SMDT1, SMEK3P, SMG6, SMIM10, SMIM9, SMN1, SMN2, SMOC1, SMOC2, SMPD1, SMPX, SMS, SNAI2, SNAP25, SNAP29, SNCA, SNCAIP, SNCB, SND1, SNIP1, SNORA11, SNORA11C, SNORA11D, SNORA11E, SNORA35, SNORA36A, SNORA56, SNORA69, SNORA70, SNORD61, SNORD96B, SNPH, SNRNP200, SNRPE, SNRPN, SNTA1, SNX10, SNX12, SOBP, SOD1, SOD2, SOS1, SOST, SOWAHD, SOX10, SOX11, SOX17, SOX18, SOX2, SOX3, SOX5, SOX9, SP110, SP7, SPACA5, SPACA5B, SPAG1, SPANXA1, SPANXA2, SPANXA2-OT1, SPANXB1, SPANXC, SPANXD, SPANXN1, SPANXN2, SPANXN3, SPANXN4, SPANXN5, SPAST, SPATA16, SPATA7, SPG11, SPG20, SPG21, SPG7, SPIN2A, SPIN2B, SPIN3, SPIN4, SPINK1, SPINK5, SPINT2, SPP1, SPR, SPRED1, SPRY4, SPRYD4, SPTA1, SPTAN1, SPTB, SPTBN2, SPTLC1, SPTLC2, SQRDL, SQSTM1, SRC, SRCAP, SRD5A1P1, SRD5A2, SRD5A3, SRP72, SRPK3, SRPX, SRPX2, SRY, SSBP1, SSR4, SSX1, SSX2, SSX2B, SSX3, SSX4, SSX4B, SSX5, SSX6, SSX7, SSX8, ST14, ST3GAL3, ST3GAL4, ST3GAL5, ST7, STAC3, STAG2, STAG3, STAMBP, STAR, STARD8, STAT1, STAT3, STAT4, STAT5A, STAT5B, STC2, STEAP3, STIL, STIM1, STK11, STK3, STK4, STOML2, STRA6, STRADA, STRC, STS, STT3A, STT3B, STUB1, STX11, STX16, STXBP1, STXBP2, SUCLA2, SUCLG1, SUCLG2, SUFU, SUGCT, SULF1, SULT1A1, SUMF1, SUMO1, SUOX, SUPT20HL1, SUPT20HL2, SUPV3L1, SURF1, SUV39H1, SYAP1, SYCP3, SYN1, SYNE1, SYNE2, SYNE4, SYNGAP1, SYP, SYP-AS1, SYT14, SYTL4, SYTL5, SZT2, T, TAAR6, TAB3, TAC3, TACO1, TACR3, TACSTD2, TAF1, TAF2, TAF4B, TAF7L, TAF9B, TAGAP, TALDO1, TAMM41, TAP1, TAP2, TAPBP, TARDBP, TARS2, TAT, TATDN3, TAZ, TBC1D20, TBC1D24, TBC1D25, TBC1D4, TBC1D8B, TBCA, TBCE, TBL1X, TBP, TBRG4, TBX1, TBX15, TBX19, TBX20, TBX22, TBX3, TBX4, TBX5, TBX6, TBXA2R, TBXAS1, TCAIM, TCAP, TCEAL1, TCEAL2, TCEAL3, TCEAL4, TCEAL5, TCEAL6, TCEAL7, TCEAL8, TCEANC, TCF12, TCF21, TCF4, TCIRG1, TCN1, TCN2, TCOF1, TCP11X2, TCTN1, TCTN2, TCTN3, TDGF1, TDGF1P3, TDP1, TDRD7, TDRKH, TEAD1, TECPR2, TECR, TECTA, TEK, TEKT2, TENM1, TERC, TERT, TET2, TEX11, TEX13A, TEX13B, TEX28, TF, TFAM, TFAP2A, TFAP2B, TFB1M, TFB2M, TFDP3, TFE3, TFG, TFR2, TG, TGFB1, TGFB2, TGFB3, TGFBI, TGFBR1, TGFBR2, TGFBR3, TGIF1, TGIF2LX, TGM1, TGM5, TGM6, TH, THAP1, THBD, THEM4, THEM5, THG1L, THOC2, THPO, THRA, THRB, TIA1, TIMM10, TIMM10B, TIMM13, TIMM17A, TIMM17B, TIMM21, TIMM22, TIMM23, TIMM44, TIMM50, TIMM8A, TIMM8B, TIMM9, TIMMDC1, TIMP1, TIMP3, TINF2, TJP2, TK1, TK2, TKTL1, TLL1, TLR3, TLR4, TLR7, TLR8, TLR8-AS1, TMC1, TMC6, TMC8, TMCO1, TMEM11, TMEM126A, TMEM126B, TMEM127, TMEM138, TMEM143, TMEM14C, TMEM160, TMEM164, TMEM165, TMEM185A, TMEM186, TMEM187, TMEM216, TMEM223, TMEM231, TMEM237, TMEM240, TMEM255A, TMEM257, TMEM27, TMEM31, TMEM35, TMEM38A, TMEM38B, TMEM43, TMEM47, TMEM5, TMEM65, TMEM67, TMEM70, TMIE, TMLHE, TMLHE-AS1, TMPO, TMPRSS15, TMPRSS3, TMPRSS5, TMPRSS6, TMSB15A, TMSB15B, TMSB4X, TMTC1, TNC, TNFAIP3, TNFRSF11A, TNFRSF11B, TNFRSF13B, TNFRSF13C, TNFRSF14, TNFRSF1A, TNFRSF4, TNFSF11, TNFSF4, TNMD, TNNC1, TNNI1, TNNI2, TNNI3, TNNT1, TNNT2, TNNT3, TNPO3, TNXB, TOMM20, TOMM22, TOMM34, TOMM40, TOMM40L, TOMM5, TOMM7, TOMM70A, TOP1MT, TOP2A, TOPORS, TOR1A, TOR1AIP1, TP53, TP63, TP73, TPH1, TPH2, TPI1, TPK1, TPM1, TPM2, TPM3, TPMT, TPO, TPP1, TPRN, TRAF1, TRAF3, TRAF3IP2, TRAF6, TRAP1, TRAPPC10, TRAPPC11, TRAPPC2, TRAPPC9, TRDN, TREM2, TREX1, TREX2, TRH, TRHR, TRIAP1, TRIB1, TRIM2, TRIM32, TRIM37, TRIOBP, TRIP11, TRIT1, TRMT10C, TRMT2B, TRMU, TRNT1, TRO, TRPA1, TRPC5, TRPC5OS, TRPC6, TRPM1, TRPM4, TRPM6, TRPM7, TRPM8, TRPS1, TRPV1, TRPV3, TRPV4, TSC1, TSC2, TSC22D3, TSEN2, TSEN34, TSEN54, TSFM, TSHB, TSHR, TSHZ1, TSHZ3, TSIX, TSPAN12, TSPAN6, TSPAN7, TSPEAR, TSPO, TSPYL1, TSPYL2, TSR2, TST, TTBK2, TTC19, TTC21B, TTC37, TTC39B, TTC3P1, TTC8, TTI2, TTLL5, TTN, TTPA, TTR, TUBA1A, TUBA8, TUBB2B, TUBB3, TUBB4A, TUBG1, TUBGCP6, TUFM, TULP1, TUSC3, TWIST1, TWIST2, TXLNG, TXN, TXN2, TXNRD1, TXNRD2, TYK2, TYMP, TYMS, TYR, TYROBP, TYRP1, UBA1, UBE2A, UBE2C, UBE2DNL, UBE2E4P, UBE2NL, UBE3A, UBIAD1, UBL4A, UBQLN2, UBR1, UCHL1, UCN2, UCP1, UCP2, UCP3, UGT1A1, UGT1A4, UIMC1, UMOD, UMPS, UNC119, UNC13B, UNC13D, UNC93B1, UNG, UPB1, UPF3B, UPK2, UPK3A, UPRT, UQCC1, UQCR10, UQCR11, UQCRB, UQCRBP1, UQCRC1, UQCRC2, UQCRFS1, UQCRH, UQCRQ, UROC1, UROD, UROS, USB1, USF1, USH1C, USH1G, USH2A, USMG5, USP11, USP24, USP26, USP27X, USP27X-AS1, USP51, USP9X, USP9Y, UTP14A, UTP14C, UTRN, UTY, UVSSA, UXS1, UXT, UXT-AS1, VAMP1, VAMP2, VAMP8, VANGL1, VANGL2, VAPB, VARS2, VAT1, VAV1, VAX1, VBP1, VCAN, VCL, VCP, VCX, VCX2, VCX3A, VCX3B, VDAC1, VDAC2, VDAC3, VDR, VEGFA, VENTXP1, VGLL1, VHL, VIM, VIPAS39, VKORC1, VLDLR, VMA21, VPS13A, VPS13B, VPS33B, VPS35, VPS37A, VPS45, VRK1, VRK2, VSIG1, VSIG4, VSX1, VSX2, VWF, WARS2, WAS, WBP5, WBSCR16, WDPCP, WDR11, WDR12, WDR13, WDR19, WDR34, WDR35, WDR36, WDR44, WDR45, WDR60, WDR62, WDR72, WDR81, WFS1, WIPF1, WIPF2, WISP3, WNK1, WNK3, WNK4, WNT1, WNT10A, WNT10B, WNT2, WNT3, WNT4, WNT5A, WNT7A, WRAP53, WRN, WT1, WWC3, WWOX, XDH, XGY2, XIAP, XIST, XK, XKR6, XKRX, XPA, XPC, XPNPEP2, XPNPEP3, XRCC1, XRCC2, XRCC3, XRCC4, XRCC6P5, XYLT1, YARS, YARS2, YBEY, YIPF6, YME1L1, YWHAE, YY2, ZADH2, ZAP70, ZBTB16, ZBTB24, ZBTB33, ZC3H12B, ZC3H14, ZC4H2, ZCCHC12, ZCCHC13, ZCCHC16, ZCCHC18, ZCCHC5, ZDHHC15, ZDHHC9, ZEB1, ZEB2, ZFP57, ZFP92, ZFPM2, ZFX, ZFX-AS1, ZFYVE20, ZFYVE26, ZFYVE27, ZIC1, ZIC2, ZIC3, ZIC4, ZMAT1, ZMPSTE24, ZMYM3, ZMYND10, ZMYND11, ZMYND15, ZNF157, ZNF182, ZNF185, ZNF275, ZNF280C, ZNF335, ZNF33A, ZNF407, ZNF41, ZNF423, ZNF449, ZNF469, ZNF507, ZNF513, ZNF526, ZNF592, ZNF630, ZNF630-AS1, ZNF645, ZNF674, ZNF674-AS1, ZNF711, ZNF75D, ZNF804A, ZNF81, ZNF90, ZNHIT6, ZPR1, ZRSR2, ZXDA, ZXDB,

**4. Patients diagnosed by AADSM**

| **Sample Name** | **Mutation** | **Final Diagnosis** | **Rank of the clearly diagnosed disease in all reported diseases** |
| --- | --- | --- | --- |
| WHP3 | GJA3(NM_021954.3):c.199G>C(p.D67H) | Cataract 14 | 1 |
| WHP9 | BBS1(NM_024649.4):c.1772C>T(p.A591V)/c.1121C>G(p.T374S) | Bardet-Biedl Syndrome 1 | 60 |
| WHP10 | FCN3(NM_003665.2):c.349delC(p.L117Sfs*65)/c.498G>C(p.E166D) | Immunodeficiency due to ficolin 3 deficiency | 26 |
| WHP11 | TCIRG1 (NM_006019.3):c.1555-2A>C /c.1775G>A(p.W592*) | Autosomal Recessive Osteopetrosis 1 | 1 |
| WHP14 | IFT140 (NM_014714.3):c.1219C>T(p.R407W)/c.2446C>T(p.R816W) | Short-rib thoracic dysplasia 9 with or without polydactyly | 24 |
| WHP22 | SLC2A10(NM_030777.3):c.685C>T(p.R229*)/c.752T>C(p.L251P) | Arterial tortuosity syndrome | 43 |
| WHP23 | GJB1(NM_000166.5):c.8G>A(p.W3*) | X-linked Charcot-Marie-Tooth disease 1 | 3 |
| WHP35 | ADCK4 (NM_024876.3):c.748G>C(p.D250H)/c.737G>A(p.S246N) | Nephrotic Syndrome Type 9 | 1 |
| WHP36 | CLCN5 (NM_001127899.1):c.992_993insAGTATTAT(p.F334Xfs*1) | Dent Disease 1 | 13 |
| WHP43 | COL4A4(NM_000092.4):c.4333G>A(p.G1445R)/c.1505delC(p.P502Lfs*151) | Alport Syndrome | 1 |
| WHP45 | COL4A5(NM_033380.2):c.3817G>T(p.G1273C) | X-linked Alport Syndrome | 1 |
| WHP46 | COL4A5(NM_033380.2):c.901G>C(p.G301R) | X-linked Alport Syndrome | 7 |
| WHP48 | CRYBB2 (NM_000496.2):c.107_115delGGCCCTGCCinsCGAGTTTCCAACCTGAAGTTT | Cataract 3 | 3 |
| WHP51 | ANLN(NM_018685.2):c.3062A>T (p.D1021V) | Focal Segmental Glomerulosclerosis 8 | 4 |
| WHP53 | CRYBB2(NM_000496.2):c.487C>T(p.Q163*) | Cataract 3 | 1 |
| WHP54 | BFSP2(NM_003571.2):c.113G>A(p.S38N) | Cataract 12 | 4 |
| WHP56 | COL4A4 (NM_000092.4):c.4214_4215insC(p.G1406Rfs*27) | Alport Syndrome | 1 |
| WHP57 | INF2 (NM_022489.3):c.1978C>T(p.R660W); COL4A4 (NM_000092.4):c.3647G>C(p.G1216A); CFH(NM_000186.3):c.3566T>G(p.L1189R) | Focal Segmental Glomerulosclerosis 5; Alport Syndrome; Hemolytic Uremic syndrome susceptibility to 1 | 1 |
| WHP58 | COL4A5(NM_033380.2):c.232-6_244delTTTCAGGGTGATGATGGAA | X-linked Alport Syndrome | 3 |
| WHP67 | BBS2(NM_031885.3)c.647G>C (p.R216P)/c.534+1G>T | Bardet-Biedl Syndrome 2 | 1 |
| WHP68 | CNGA1(NM_001142564.1):c.829G>A (p.D277N)/c.472delC(p.L158Ffs*4) | Retinitis pigmentosa 49 | 1 |
| WHP74 | IQCB1(NM_001023570.2):c.1090C>T(p.R364*)/c.1225C>T(p.Q409*) | Senior-Loken syndrome 5 | 1 |
| WHP81 | WT1(NM_024426.4) :c.1432+5G>A | Denys-Drash syndrome; Meacham Syndrome; Frasier syndrome | 10 |
| WHP82 | LONP1(NM_004793.2):c.2392G>A(p.G798S)/c.2290G>A(p.V764M) | CODAS Syndrome | 106 |
| WHP95 | CBS (NM_001178009.1):c.526G>A(p.E176K)/c.949A>G(p.R317G) | Homocystinuria Caused by Cystathionine Beta-Synthase Deficiency | 1 |
| WHP96 | CFH (NM_000186.3):c.3643C>G(p.R1215G); CD46(NM_172359.2):c.1114A>T(p.R372W) | Hemolytic Uremic syndrome susceptibility to 1; Hemolytic Uremic syndrome susceptibility to 2 | 6 |
| WHP101 | TSC2(NM_000548.3):c.977delC(p.M327Wfs*36) | Tuberous Sclerosis 2 | 1 |
| WHP109 | NOTCH3(NM_000435.2):c.505C>T(R169C) | Cerebral Autosomal Dominant Arteriopathy with Subcortical Infarcts And Leukoencephalopathy Type 1 | 1 |
| WHP110 | PPT1(NM_000310.3):c.722C>T(p.S241L)/CDS9 deletion | Neuronal Ceroid-Lipofuscinoses 1 | 28 |
| WHP111 | MMACHC(NM_015506.2):c.82-2A>G/c.463G>C(p.G155R) | Methylmalonic aciduria and homocystinuria CblC type | 1 |
| WHP115 | COL4A5(NM_033380.2):c.262C>T(p.P88S) | X-linked Alport Syndrome | 11 |
| WHP117 | PKHD1(NM_138694.3):c.9455delA (p.N3152Tfs*10)/c.5231A>G(p.N1744S) | Autosomal Recessive Polycystic Kidney Disease | 2 |
| WHP118 | TTC21B(NM_024753.4):c.1552T>C(p.C518R)/c.895T>C(p.C299R) | Nephronophthisis 12 | 38 |
| WHP119 | PMP22(NM_153322.1):Whole gene duplication | Charcot-Marie-Tooth disease | 1 |
| WHP123 | COL4A3(NM_000091.4):c.1106G>A(p.G369D) | Alport Syndrome | 1 |
| WHP124 | CFHR1(NM_002113.2):CDS2-6 deletion;CFHR3(NM_021023.5):Whole gene duplication | Hemolytic Uremic syndrome susceptibility to 1 | 1 |
| WHP125 | SLC12A3 (NM_000339.2):c.1856G>A (p.G619D)/c.2451_2458delCCCCAAGG(p.K819Gfs*26) | Gitelman syndrome | 1 |
| WHP126 | COL4A5(NM_033380.2):c.5038C>T(p.R1680*) | X-linked Alport Syndrome | 1 |
| WHP127 | NOTCH3(NM_000435.2):c.472G>A(p.D158N) | Cerebral Autosomal Dominant Arteriopathy with Subcortical Infarcts And Leukoencephalopathy Type 1 | 1 |
| WHP128 | MKS1(NM_017777.3): c.1601G>A(p.R534Q)/c.323G>A(p.R108H) | Joubert Syndrome 28; Bardet-Biedl Syndrome 13 | 33 |
| WHP129 | PAX2(NM_003990.3):c.418C>T( p.R140W ) | Focal Segmental Glomerulosclerosis 7 | 4 |
| WHP131 | NOTCH3(NM_000435.2):c.1010A>G(p.Y337C) | Cerebral Autosomal Dominant Arteriopathy with Subcortical Infarcts And Leukoencephalopathy Type 1 | 1 |
| WHP132 | TTC21B(NM_024753.4):c.1897C>T(p.Q633* )/c.1552T>C(p.C518R ) | Nephronophthisis 12 | 1 |
| WHP133 | IMPAD1(NM_017813.4):c.700G>T (p.E234*)/CDS4-5 deletion | Chondrodysplasia with Joint Dislocations | 1 |
| WHP134 | COL4A5(NM_033380.2):c.465+5G>T | X-linked Alport Syndrome | 3 |
| WHP135 | COL4A4(NM_000092.4):c.2311G>A(p.G771R) | Alport Syndrome | 5 |
| WHP137 | AVPR2(NM_000054.4):c.409C>T(p.R137C) | Nephrogenic Syndrome of Inappropriate Antidiuresis | 1 |
| WHP138 | SLC26A3(NM_000111.2):c.386C>T(p.P129L )/c.270-271insAA(p.G91Kfs*3 ) | Familial Chloride Diarrhea | 1 |
| WHP139 | COL4A3(NM_000091.4):c.4664C>T( p.A1555V); COL4A4(NM_000092.4):c.870G>T(p.K290N ) | Alport Syndrome | 1 |
| WHP140 | AGXT(NM_000030.2):c.1015delG(p.V339Sfs*2) | Primary Hyperoxaluria Type 1 | 1 |
| WHP141 | SLC12A1(NM_000338.2):c.463G>A(p.G155S)/c.3207G>T(p.L1069F) | Bartter Syndrome 1 | 2 |
| WHP146 | COL4A5(NM_033380.2): c.3125G>T(p.G1042V) | X-linked Alport Syndrome | 1 |
| WHP148 | GAA(NM_000152.3):c.953T>A(p.M318K)/c.2184delC(p.L729Wfs*35) | Glycogen storage disease II | 1 |
| WHP150 | LPIN1(NM_145693.2):c.357_358insCT(p.K121Terfs*1) | Acute Recurrent Myoglobinuria | 1 |
| WHP151 | FANCI(NM_001113378.1):c.286G>A(p.E96K)/c.3457C>G(p.L1153V) | Fanconi anemia | 54 |
| WHP152 | RS1(NM_000330.3):c.214G>A | X-Linked Juvenile Retinoschisis | 1 |

**5. Population frequencies of the identified variants**

| Sample Name |  |  | Mutation Information |  | Frequency in 1k Genome | Frequency in gnomAD | Frequency in esp6500 | Frequency in ExAC |
| --- | --- | --- | --- | --- | --- | --- | --- | --- |
|  | Gene | NM Number | CDS | Amino acid |  |  |  |  |
| WHP1 | PYGM | NM_005609.2 | c.1948C>T | p.R650* | - | - | - | 8.25E-06 |
| WHP2 | GJA3 | NM_021954.3 | c.199G>C | p.D67H | - | - | - | - |
| WHP3 | GJA3 | NM_021954.3 | c.199G>C | p.D67H | - | - | - | - |
| WHP4 | MIP | NM_012064.3 | c.605G>A | p.W202* | - | - | - | - |
| WHP5 | HSF4 | NM_001040667.2 | c.179C>T | p.P60H | - | 4.07E-06 | - | - |
| WHP6 | EVC | NM_153717.2 | c.1436C>T | p.P479L | - | 3.66E-05 | - | 8.25E-06 |
| WHP7 | CFHR1  CFHR3 | NM_002113.2  NM_021023.5 | whole genes deletion  whole genes deletion |  | N.A.  N.A. | N.A.  N.A. | N.A.  N.A. | N.A.  N.A. |
| WHP8 | ATP6V0A4  ATP6V0A4 | NM_020632.2  NM_020632.2 | c.1180G>A  c.620_621insC | p.A394T  p.L208Sfs*33 | -  - | -  - | -  - | -  - |
| WHP9 | BBS1  BBS1 | NM_024649.4  NM_024649.4 | c.1772C>T  c.1121C>G | p.A591V  p.T374S | 0.0002  - | 0.0001  8.12E-06 | 0.0002  - | 0.0003  8.24E-06 |
| WHP10 | FCN3  FCN3 | NM_003665.2  NM_003665.2 | c.349delC  c.498G>C | p.L117Sfs*65  p.E166D | -  0.005192 | -  0.0024 | -  0.0002 | -  0.0026 |
| WHP11 | TCIRG1  TCIRG1 | NM_006019.3  NM_006019.3 | c.1555-2A>C  c.1775G>A | p.W592* | -  - | 2.05E-05  - | -  - | 3.31E-05  - |
| WHP12 | PAX6 | NM_001258462.1 | c.120C>A | p.C40* | - | - | - | - |
| WHP13 | HSF4 | NM_001040667.2 | c.558C>T | p.G186G | - | 4.25E-06 | - | - |
| WHP14 | IFT140  IFT140 | NM_014714.3  NM_014714.3 | c.1219C>T  c.2446C>T | p.R407W  p.R816W | -  0.0002 | 7.40E-05  0.0003 | -  - | 9.35E-05  0.0003 |
| WHP15 | COL4A4  TSC1 | NM_000092.4  NM_000368.4 | c.1624-1G>A  c.1460C>G | p.S487C | -  0.0002 | -  0.0004 | -  0.0002 | -  0.0005 |
| WHP16 | MIP | NM_012064.3 | c.494G>A | p.G165D | - | - | - | - |
| WHP17 | PMP22  MYH14 | NM_153322.1  NM_001145809.1 | c.319+1G>T  c.3040G>C | p.E1014Q | -  - | -  - | -  - | -  - |
| WHP18 | IMPDH1 | NM_000883.3 | c.626C>T | p.S209L | 0.0002 | 9.44E-05 | - | 4.43E-05 |
| WHP19 | IMPDH1 | NM_000883.3 | c.626C>T | p.S209L | 0.0002 | 9.44E-05 | - | 4.43E-05 |
| WHP20 | VCAN | NM_004385.4 | c.7870G>A | p.E2624K | - | 1.22E-05 | - | 1.65E-05 |
| WHP21 | LMNB1 | NM_005573.3 | c.1365C>T | p.R455R | - | 8.16E-06 | - | 8.30E-06 |
| WHP22 | SLC2A10  SLC2A10 | NM_030777.3  NM_030777.3 | c.685C>T  c.752T>C | p.R229*  p.L251P | -  - | 1.63E-05  - | -  - | 3.31E-05  - |
| WHP23 | GJB1 | NM_000166.5 | c.8G>A | p.W3* | - | - | - | - |
| WHP24 | ATP6V1B1  ATP6V1B1 | NM_001692.3  NM_001692.3 | c.368-1G>A  c.1354delT | p.F452Lfs*35 | -  - | -  - | -  - | -  - |
| WHP25 | GUSB | NM_000181.3 | c.1192C>T | p.R398C | - | 2.44E-05 | - | 5.77E-05 |
| WHP26 | RHO | NM_000539.3 | c.403C>T | p.R135W | - | - | - | - |
| WHP27 | RP1 | NM_006269.1 | c.1437G>T | p.M479I | - | 4.06E-05 | - | 6.60E-05 |
| WHP28 | USH2A  USH2A | NM_206933.2  NM_206933.2 | c.13465G>A  c.8641_8642insTATT | p.G4489S  p.S2881Lfs*9 | -  - | -  - | -  - | -  - |
| WHP29 | USH2A  USH2A | NM_206933.2  NM_206933.2 | c.9958G>T  c.99_100insT | p.G3320C  p.R34Sfs*41 | -  - | -  - | -  - | -  - |
| WHP30 | OCRL  SLC7A9 | NM_000276.3  NM_001243036.1 | c.1040G>A  c.829G>A | p.G347E  p.V277M | -  0.000998 | -  0.0006 | -  0.0002 | -  0.0005 |
| WHP31 | SLC12A3  SLC12A3 | NM_000339.2  NM_000339.2 | c.1362_1363insC  c.2029G>A | p.L457Dfs*68  p.V677M | -  - | -  1.22E-05 | -  - | -  8.31E-06 |
| WHP32 | ARHGEF10  PMP22 | NM_014629.2  NM_153322.1 | c.824G>A  whole gene duplication | p.R275H | 0.002596  N.A. | 0.0013  N.A. | -  N.A. | 0.0012  N.A. |
| WHP33 | EPHA2  MYOC | NM_004431.3  NM_000261.1 | c.944G>A  c.1432G>T | p.R315Q  p.D478Y | 0.0002  - | 0.0014  - | 0.0017  - | 0.0016  - |
| WHP34 | NLRP3 | NM_004895.4 | c.214G>A | p.V72M | 0.002196 | 0.0007 | - | 0.0006 |
| WHP35 | ADCK4  ADCK4 | NM_024876.3  NM_024876.3 | c.748G>C  c.737G>A | p.D250H  p.S246N | -  0.0002 | 4.51E-05  6.54E-05 | -  - | 2.51E-05  9.19E-05 |
| WHP36 | CLCN5 | NM_001127899.1 | c.992_993insAGTATTAT | p.F334Xfs*1 | - | - | - | - |
| WHP37 | GJA8 | NM_005267.4 | c.154T>C | p.F52L | - | - | - | - |
| WHP38 | GJA3 | NM_021954.3 | c.1152_1153insG | p.S385Efs*83 | - | - | - | - |
| WHP39 | CRYBB2 | NM_000496.2 | c.152C>T | p.S51F | - | - | - | - |
| WHP40 | ATP7B  ATP7B | NM_000053.3  NM_000053.3 | c.2333G>T  c.2310C>G | p.R778L  p.L770L | -  - | 0.0001  0.0001 | -  - | 0.0002  0.0002 |
| WHP41 | MPZ  DYNC1H1 | NM_000530.6  NM_001376.4 | c.286A>C  c.12804C>T | p.K96Q  p.F4268F | -  - | -  1.22E-05 | -  - | -  1.65E-05 |
| WHP42 | CFHR5 | NM_030787.3 | c.508G>A | p.V170M | 0.001198 | 0.0006 | - | 0.0006 |
| WHP43 | COL4A4  COL4A4 | NM_000092.4  NM_000092.4 | c.4333G>A  c.1505delC | p.G1445R  p.P502Lfs*151 | -  - | -  - | -  - | -  - |
| WHP44 | CYP4V2 | NM_207352.3 | c.802-6_810delATACAGGTCATCGCT |  | - | - | - | - |
| WHP45 | COL4A5 | NM_033380.2 | c.3817G>T | p.G1273C | - |  | - | - |
| WHP46 | COL4A5 | NM_033380.2 | c.901G>C | p.G301R | - | -. | - | - |
| WHP47 | MFN2 | NM_014874.3 | c.385A>G | p.T129A | - | - | - | - |
| WHP48 | CRYBB2 | NM_000496.2 | c.107_115delGGCCCTGCCinsCGAGTTTCCAACCTGAAGTTT |  | - | - | - | - |
| WHP49 | MYOC | NM_000261.1 | c.136C>T | p.R46* | 0.001398 | 0.0007 | - | 0.0007 |
| WHP50 | EXT1 | NM_000127.2 | c.957T>G | p.Y319* | - | - | - | - |
| WHP51 | ANLN | NM_018685.2 | c.3062A>T | p.D1021V | - | 1.69E-05 | - | - |
| WHP52 | GJA8 | NM_005267.4 | c.134G>C | p.W45S | - | - | - | - |
| WHP53 | CRYBB2 | NM_000496.2 | c.487C>T | p.Q163* | - | - | - | - |
| WHP54 | BFSP2 | NM_003571.2 | c.113G>A | p.S38N | - | 4.07E-06 | - | 8.27E-06 |
| WHP55 | CRYBB2 | NM_000496.2 | c.463C>T | p.Q155* | - | - | - | - |
| WHP56 | COL4A4 | NM_000092.4 | c.4214_4215insC | p.G1406Rfs*27 | - | - | - | - |
| WHP57 | INF2  COL4A4  CFH | NM_022489.3  NM_000092.4  NM_000186.3 | c.1978C>T  c.3647G>C  c.3566T>G | p.R660W  p.G1216A  p.L1189R | 0.001997  -  - | 0.0001  -  - | -  -  - | 0.0003  -  - |
| WHP58 | COL4A5 | NM_033380.2 | c.232-6_244delTTTCAGGGTGATGATGGAA |  | - | - | - | - |
| WHP59 | NTRK1  NTRK1 | NM_002529.3  NM_002529.3 | c.632T>A  c.1253_1254delTC | p.V211E  p.S419Gfs*80 | -  - | -  - | -  - | -  - |
| WHP60 | TDRD7  CRYBA2 | NM_014290.2  NM_057094.1 | c.688_689insA  c.457T>C | p.Y230Terfs*1  p.Y153H | -  0.0002 | -  0.0001 | -  - | -  0.0001 |
| WHP61 | OCRL | NM_000276.3 | CDS20-23 deletion |  | N.A. | N.A. | N.A. | N.A. |
| WHP62 | AGXT  AGXT | NM_000030.2  NM_000030.2 | c.332G>A  c.815_816insGA | p.R111Q  p.S275Rfs*38 | -  - | 2.92E-05  - | 7.70E-05  - | 3.47E-05  - |
| WHP63 | PAX6 | NM_001258462.1 | c.817_818insT | p.S273Ffs*2 | - | - | - | - |
| WHP64 | FBN2  MYH8 | NM_001999.3  NM_002472.2 | c.4217G>A  c.4288G>T | p.C1406Y  p.D1430Y | -  - | -  2.03E-05 | -  - | -  8.24E-06 |
| WHP65 | CLCN5 | NM_001127899.1 | c.1272delG | p.K424Nfs*5 | - | - | - | - |
| WHP66 | BBS2  BBS2 | NM_031885.3  NM_031885.3 | c.943C>T  c.534+1G>T | p.R315W | -  - | 1.63E-05  9.75E-05 | -  - | 1.65E-05  0.0001 |
| WHP67 | BBS2  BBS2 | NM_031885.3  NM_031885.3 | c.647G>C  c.534+1G>T | p.R216P | -  - | 4.07E-06  9.75E-05 | 7.70E-05  - | 8.29E-06  0.0001 |
| WHP68 | CNGA1  CNGA1 | NM_001142564.1  NM_001142564.1 | c.829G>A  c.472delC | p.D277N  p.L158Ffs*4 | -  - | -  0.0001 | -  - | -  9.13E-05 |
| WHP69 | IGHMBP2  IGHMBP2 | NM_002180.2  NM_002180.2 | c.344C>T  CDS10-14 duplication | p.T115M | 0.002396  N.A. | 0.0009  N.A. | 7.70E-05  N..A | 0.0009  N.A. |
| WHP70 | EPHA2 | NM_004431.3 | c.983C>A | p.P328H | - | - | - | - |
| WHP71 | XPA  XPA | NM_000380.3  NM_000380.3 | c.283G>A  c.631C>T | p.G95R  p.R211* | -  - | -  4.07E-06 | -  7.70E-05 | -  8.26E-06 |
| WHP72 | RP1L1 | NM_178857.5 | c.32C>T | p.P11L | 0.000799 | 0.0008 | 0.0013 | 0.0008 |
| WHP73 | GJA8 | NM_005267.4 | c.163A>G | p.N55D | - | - | - | - |
| WHP74 | IQCB1  IQCB1 | NM_001023570.2  NM_001023570.2 | c.1090C>T  c.1225C>T | p.R364*  p.Q409* | -  - | 4.88E-05  - | -  - | 4.94E-05  - |
| WHP75 | USH2A  USH2A | NM_206933.2  NM_206933.2 | c.4165delG  c.11156G>A | p.V1389Lfs*43  p.R3719H | -  - | -  5.28E-05 | -  - | -  5.77E-05 |
| WHP76 | CRYBB3 | NM_004076.3 | c.593G>A | p.R198H | 0.000399 | 7.34E-05 | - | 7.64E-05 |
| WHP77 | SLC12A3 | NM_000339.2 | c.468C>A | p.Y156* | - | - | - | - |
| WHP78 | FLG  FLG | NM_002016.1  NM_002016.1 | c.5841G>A  c.3321delA | p.W1947*  p.G1109Efs*13 | -  0.001398 | 2.84E-05  0.0007 | -  8.00E-05 | 3.30E-05  0.0007 |
| WHP79 | FLG | NM_002016.1 | c.3321delA | p.G1109Efs*13 | 0.001398 | 0.0007 | 8.00E-05 | 0.0007 |
| WHP80 | CRYBB3 | NM_004076.3 | c.466G>A | p.G156R | - | - | - | - |
| WHP81 | WT1 | NM_024426.4 | c.1432+5G>A |  | - | - | - | - |
| WHP82 | LONP1  LONP1 | NM_004793.2  NM_004793.2 | c.2392G>A  c.2290G>A | p.G798S  p.V764M | 0.002796  - | 0.0009  8.15E-06 | -  7.70E-05 | 0.0009  8.33E-06 |
| WHP83 | FLG | NM_002016.1 | c.3905C>A | p.S1302* | - | 0.0001 | - | 9.06E-05 |
| WHP84 | FLG | NM_002016.1 | c.3905C>A | p.S1302* | - | 0.0001 | - | 9.06E-05 |
| WHP85 | FLG | NM_002016.1 | c.3905C>A | p.S1302* | - | 0.0001 | - | 9.06E-05 |
| WHP86 | NHS | NM_198270.2 | c.985G>T | p.E329* | - | - | - | - |
| WHP87 | CFB | NM_001710.5 | c.1598A>G | p.K533R | 0.019169 | 0.0092 | 0.0044 | 0.0105 |
| WHP88 | COL4A5 | NM_033380.2 | c.2215C>G | p.P739A | 0.003444 | 0.0011 | - | 0.0028 |
| WHP89 | GJA8 | NM_005267.4 | c.773C>T | p.S258F | - | - | - | - |
| WHP90 | ALDH7A1  ALDH7A1 | NM_001182.4  NM_001182.4 | c.1279G>C  CDS8-13 deletion | p.E427Q | 0.0002  N.A. | 0.0004  N.A. | 0.0005  N.A. | 0.0004  N.A. |
| WHP91 | SCN9A | NM_002977.3 | c.4174-1G>C |  | - | - | - | - |
| WHP92 | SCN9A  SCN9A | NM_002977.3  NM_002977.3 | c.850delG  c.129_141delTGAAGAAGCCCCA | p.E284Kfs*3  p.D43Efs*43 | -  - | -  - | -  - | -  - |
| WHP93 | SCN9A  SCN9A | NM_002977.3  NM_002977.3 | c.296G>A  c.2749T>G | p.R99H  p.W917G | -  - | 4.07E-06  - | -  - | -  - |
| WHP94 | SCN9A | NM_002977.3 | c.2697G>A | p.M899I | 0.0002 | 4.07E-06 | - | 8.24E-06 |
| WHP95 | CBS  CBS | NM_001178009.1  NM_001178009.1 | c.526G>A  c.949A>G | p.E176K  p.R317G | -  - | 0  8.13E-06 | -  - | -  8.26E-06 |
| WHP96 | CFH  CD46 | NM_000186.3  NM_172359.2 | c.3643C>G  c.1114A>T | p.R1215G  p.R372W | -  - | -  - | -  - | -  - |
| WHP97 | RHBDF2 | NM_024599.5 | c.566C>T | p.P189L | - | - | - | - |
| WHP98 | AVPR2 | NM_000054.4 | whole gene deletion |  | N.A. | N.A. | N.A. | N.A. |
| WHP99 | IFT172  IFT172 | NM_015662.1  NM_015662.1 | c.2053C>T  c.5071T>C | p.R685*  p.Y1691H | -  - | 1.63E-05  4.07E-06 | -  - | 8.24E-06  - |
| WHP100 | GSN  COL4A4  TNXB | NM_000177.4  NM_000092.4  NM_019105.6 | c.444G>T  c.930+1G>A  c.8201_8202insC | p.E148D  p.E2735Terfs*1 | -  -  - | 8.13E-06  -  - | -  -  - | 1.66E-05  -  - |
| WHP101 | TSC2 | NM_000548.3 | c.977delC | p.M327Wfs*36 | - | - | - | - |
| WHP102 | EYS  EYS | NM_001142800.1  NM_001142800.1 | c.8545C>T  c.5644+5G>A | p.R2849* | -  - | -  3.16E-05 | -  - | -  - |
| WHP103 | ABCA4  ABCA4 | NM_000350.2  NM_000350.2 | c.4720G>T  c.6289C>T | p.E1574*  p.P2097S | -  - | 4.06E-06  - | -  - | -  - |
| WHP104 | USH2A  USH2A | NM_206933.2  NM_206933.2 | c.142_143insGA  c.2802T>G | p.K48Rfs*98  p.C934W | -  0.000799 | -  0.0002 | -  - | -  0.0002 |
| WHP105 | CFB | NM_001710.5 | c.1697A>C | p.E566A | 0.010184 | 0.0112 | 0.0073 | 0.0111 |
| WHP106 | FBN1 | NM_000138.4 | c.385T>C | p.C129R | - | - | - | - |
| WHP107 | ZNF423  ZNF423 | NM_015069.2  NM_015069.2 | c.1801G>A  c.1537G>A | p.A601T  p.G513S | -  0.000998 | -  0.0002 | -  - | -  0.0002 |
| WHP108 | AGXT  AGXT  OCRL | NM_000030.2  NM_000030.2  NM_000276.3 | c.557C>T  c.590G>A  c.808G>A | p.A186V  p.R197Q  p.D270N | 0.026158  0.025959  - | 0.0111  0.0111  - | 0.0007  0.0007  - | 0.0114  0.0114  - |
| WHP109 | NOTCH3 | NM_000435.2 | c.505C>T | R169C | - | - | - | - |
| WHP110 | PPT1  PPT1 | NM_000310.3  NM_000310.3 | c.722C>T  CDS9 deletion | p.S241L | -  N.A. | 1.22E-05  N.A. | -  N.A. | 2.47E-05  N.A. |
| WHP111 | MMACHC  MMACHC | NM_015506.2  NM_015506.2 | c.82-2A>G  c.463G>C | p.G155R | -  - | -  - | -  - | -  - |
| WHP112 | PKD1 | NM_001009944.2 | c.7288C>T | p.R2430* | - | - | - | - |
| WHP113 | PMP22 | NM_153322.1 | Whole gene duplication |  | N.A. | N.A. | N.A. | N.A. |
| WHP114 | FBN1 | NM_000138.4 | c.5839T>C | p.C1947R | - | - | - | - |
| WHP115 | COL4A5 | NM_033380.2 | c.262C>T | p.P88S | - | 7.32E-05 | - | 0.0001 |
| WHP116 | PMP22 | NM_153322.1 | Whole gene duplication |  | N.A. | N.A. | N.A. | N.A. |
| WHP117 | PKHD1  PKHD1 | NM_138694.3  NM_138694.3 | c.9455delA  c.5231A>G | p.N3152Tfs*10  p.N1744S | -  - | -  2.85E-05 | -  - | -  2.53E-05 |
| WHP118 | TTC21B  TTC21B | NM_024753.4  NM_024753.4 | c.1552T>C  c.895T>C | p.C518R  p.C299R | -  0.0002 | -  3.34E-05 | -  - | -  3.92E-05 |
| WHP119 | PMP22 | NM_153322.1 | Whole gene duplication |  | N.A. | N.A. | N.A. | N.A. |
| WHP120 | CLCN1 | NM_000083.2 | c.847C>T | p.L283F | - | - | - | - |
| WHP121 | DCTN1 | NM_004082.4 | c.1588G>A | p.V530M | - | 4.06E-06 | - | - |
| WHP122 | AVPR2 | NM_000054.4 | whole gene deletion |  | N.A. | N.A. | N.A. | N.A. |
| WHP123 | COL4A3 | NM_000091.4 | c.1106G>A | p.G369D | - | - | - | - |
| WHP124 | CFHR1  CFHR3 | NM_002113.2  NM_021023.5 | CDS2-6 deletion;  Whole gene duplication |  | N.A.  N.A. | N.A.  N.A. | N.A.  N.A. | N.A.  N.A. |
| WHP125 | SLC12A3  SLC12A3 | NM_000339.2  NM_000339.2 | c.1856G>A  c.2451_2458delCCCCAAGG | p.G619D  p.K819Gfs*26 | -  - | -  - | -  - | -  - |
| WHP126 | COL4A5 | NM_033380.2 | c.5038C>T | p.R1680* | - | - | - | - |
| WHP127 | NOTCH3 | NM_000435.2 | c.472G>A | p.D158N | 0.0002 | 3.71E-05 | - | 3.55E-05 |
| WHP128 | MKS1  MKS1 | NM_017777.3  NM_017777.3 | c.1601G>A  c.323G>A | p.R534Q  p.R108H | 0.000799  0.000599 | 0.0002  0.0001 | -  8.40E-05 | 0.0003  0.0002 |
| WHP129 | PAX2 | NM_003990.3 | c.418C>T | p.R140W | - | 4.78E-06 | - | - |
| WHP130 | MPZ | NM_000530.6 | c.308G>C | p.G103A | - | - | - | - |
| WHP131 | NOTCH3 | NM_000435.2 | c.1010A>G | p.Y337C | - | - | - | - |
| WHP132 | TTC21B  TTC21B | NM_024753.4  NM_024753.4 | c.1897C>T  c.1552T>C | p.Q633*  p.C518R | -  - | -  - | -  - | -  - |
| WHP133 | IMPAD1  IMPAD1 | NM_017813.4  NM_017813.4 | c.700G>T  CDS4-5 deletion | p.E234* | -  N.A. | -  N.A. | -  N.A. | -  N.A. |
| WHP134 | COL4A5 | NM_033380.2 | c.465+5G>T |  | - | - | - | - |
| WHP135 | COL4A4 | NM_000092.4 | c.2311G>A | p.G771R | - | 4.06E-06 | - | - |
| WHP136 | COL4A4 | NM_000092.4 | c.4333+2T>C |  | - | 4.06E-06 | - | 8.30E-06 |
| WHP137 | AVPR2 | NM_000054.4 | c.409C>T | p.R137C | - | 5.81E-06 | - | 1.15E-05 |
| WHP138 | SLC26A3  SLC26A3 | NM_000111.2  NM_000111.2 | c.386C>T  c.270_271insAA | p.P129L  p.G91Kfs*3 | -  - | 1.63E-05  - | -  - | 2.47E-05  - |
| WHP139 | COL4A3  COL4A4 | NM_000091.4  NM_000092.4 | c.4664C>T  c.870G>T | p.A1555V  p.K290N | 0.000399  - | 4.88E-05  - | 8.10E-05  - | 4.15E-05  - |
| WHP140 | AGXT | NM_000030.2 | c.1015delG | p.V339Sfs*2 | - | - | - | - |
| WHP141 | SLC12A1  SLC12A1 | NM_000338.2  NM_000338.2 | c.463G>A  c.3207G>T | p.G155S  p.L1069F | 0.0002  - | 0.0002  0.0002 | 7.70E-05  - | 0.0002  0.0002 |
| WHP142 | SLC3A1 | NM_000341.3 | c.1332+2T>A |  | - | 8.13E-06 | - | 8.25E-06 |
| WHP143 | SETX | NM_015046.5 | c.7114G>A | p.D2372N | 0.008387 | 0.006 | 7.70E-05 | 0.0056 |
| WHP144 | CHM  GUCY2D | NM_000390.2  NM_000180.3 | c.1456_1457insG  c.2375C>T | p.A486Gfs*6  p.P792L | -  - | -  2.85E-05 | -  - | -  8.26E-06 |
| WHP145 | NTRK1  NTRK1 | NM_002529.3  NM_002529.3 | c.851-33T>A  c.1805G>A | p.R602Q | -  - | 3.45E-05  - | -  - | 3.35E-05  - |
| WHP146 | COL4A5 | NM_033380.2 | c.3125G>T | p.G1042V | - | - | - | - |
| WHP147 | SLC7A9  INF2 | NM_014270.4  NM_022489.3 | c.829G>A  c.1372C>T | p.V277M  p.P458S | 0.000998  - | 0.0006  0.0017 | 0.0002  - | 0.0005  0.0004 |
| WHP148 | GAA  GAA | NM_000152.3  NM_000152.3 | c.953T>A  c.2184delC | p.M318K  p.L729Wfs*35 | -  - | -  - | -  - | -  - |
| WHP149 | PMP22 | NM_000304.2 | CDS2-4 duplication |  | N.A. | N.A. | N.A. | N.A. |
| WHP150 | LPIN1 | NM_145693.2 | c.357_358insCT | p.K121Terfs*1 | - | - | - | - |
| WHP151 | FANCI  FANCI | NM_001113378.1  NM_001113378.1 | c.286G>A  c.3457C>G | p.E96K  p.L1153V | 0.002796  0.000399 | 0.0017  2.03E-05 | 0.0002  - | 0.0016  1.65E-05 |
| WHP152 | RS1 | NM_000330.3 | c.214G>A | p.E72K | - | 1.68E-05 | - | 1.14E-05 |

“-" means the population frequency in the database is not found.
